# Sulfur sequestration promotes multicellularity during nutrient limitation

**DOI:** 10.1038/s41586-021-03270-3

**Published:** 2021-02-24

**Authors:** Beth Kelly, Gustavo E. Carrizo, Joy Edwards-Hicks, David E. Sanin, Michal A. Stanczak, Chantal Priesnitz, Lea J. Flachsmann, Jonathan D. Curtis, Gerhard Mittler, Yaarub Musa, Thomas Becker, Joerg M. Buescher, Erika L. Pearce

**Affiliations:** 1 Max Planck Institute for Immunobiology and Epigenetics, Freiburg, Germany; 2 Institute of Biochemistry and Molecular Biology, ZMBZ, Faculty of Medicine, University of Freiburg, Freiburg, Germany; 3 Faculty of Biology, University of Freiburg, Freiburg, Germany; 4 Institute of Biochemistry and Molecular Biology, Faculty of Medicine, University of Bonn, Bonn, Germany; 5 Present Address: The Bloomberg–Kimmel Institute for Cancer Immunotherapy at Johns Hopkins, Johns Hopkins University, Baltimore, MD USA

**Keywords:** Mitochondria, Mitochondria

## Abstract

The behaviour of *Dictyostelium discoideum* depends on nutrients^1^. When sufficient food is present these amoebae exist in a unicellular state, but upon starvation they aggregate into a multicellular organism^2,3^. This biology makes *D. discoideum* an ideal model for investigating how fundamental metabolism commands cell differentiation and function. Here we show that reactive oxygen species—generated as a consequence of nutrient limitation—lead to the sequestration of cysteine in the antioxidant glutathione. This sequestration limits the use of the sulfur atom of cysteine in processes that contribute to mitochondrial metabolism and cellular proliferation, such as protein translation and the activity of enzymes that contain an iron–sulfur cluster. The regulated sequestration of sulfur maintains *D. discoideum* in a nonproliferating state that paves the way for multicellular development. This mechanism of signalling through reactive oxygen species highlights oxygen and sulfur as simple signalling molecules that dictate cell fate in an early eukaryote, with implications for responses to nutrient fluctuations in multicellular eukaryotes.

## Abstract (web summary)

Depriving unicellular *Dictyostelium discoideum* of nutrients generates reactive oxygen species that sequester cysteine within glutathione, which maintains this amoeba in a nonproliferating state that promotes aggregation into a multicellular organism.

## Main

The eukaryote *D. discoideum* bridges the unicellular-to-multicellular transition, which represents a key evolutionary step. Unicellular *D. discoideum* consume bacteria and yeast^1^; upon nutrient restriction this species aggregates into a multicellular organism, differentiating and forming a spore that regerminates in conditions favourable to growth^2^. cAMP^3^ and superoxide^4^ drive this aggregation. Superoxide and other reactive oxygen species (ROS) are common signalling molecules^5^ that influence function by oxidatively modifying proteins and modulating transcription factors^6,7^. However, excess ROS cause oxidative injury, cell death^8^ and pathology^9^. Numerous antioxidants control ROS, including superoxide dismutase, catalase and glutathione (GSH)^10^. GSH—which consists of glycine, glutamate and cysteine—has roles beyond its antioxidant function^11^, and GSH and redox status regulate normal and malignant cell proliferation^12,13^ (although the mechanism has not been fully elucidated). Here we reveal a function of ROS in increasing demand for GSH, and thus prioritizing cysteine for GSH synthesis, during nutrient restriction. This limits the sulfur supply from cysteine and thus shuts down mitochondrial metabolism and proliferation, which prompts multicellular development.

## Starvation alters mitochondrial activity

**Fig. 1:**
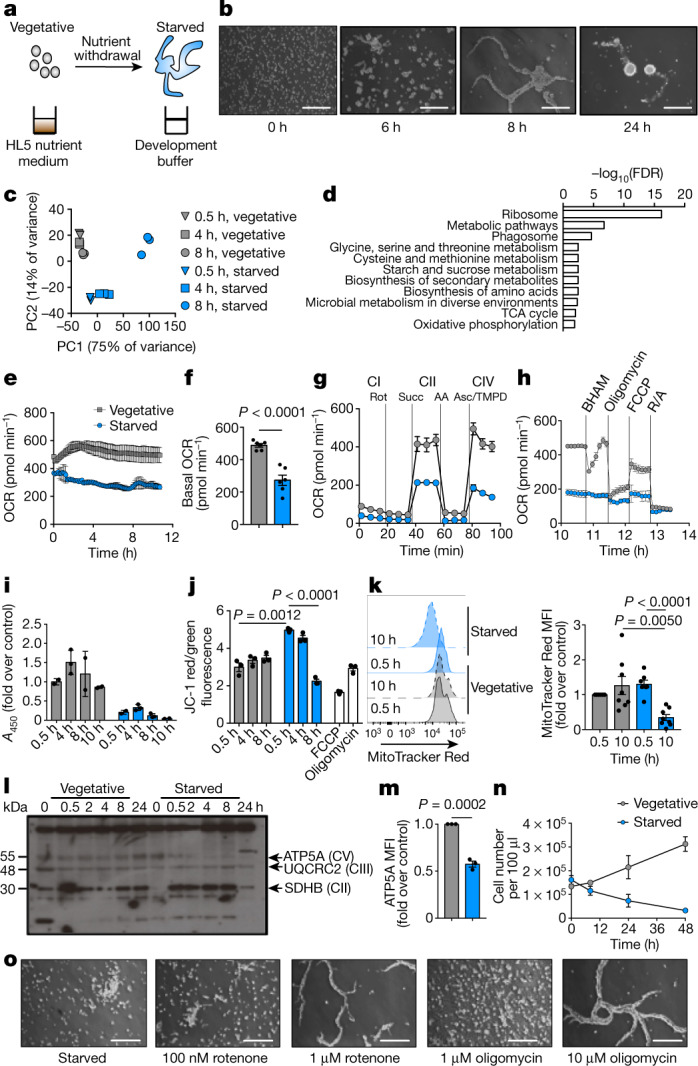
Starving *D. discoideum* decrease their mitochondrial metabolism. **a**, Experimental system for *D. discoideum* starvation. **b**, *Dictyostelium discoideum* aggregation upon starvation (*n* = 40). Scale bars, 50 μm. **c**, Principal component (PC) analysis of RNA-seq of vegetative or starved *D. discoideum* (*n* = 3). **d**, RNA-seq pathway analysis, showing the most significantly regulated pathways in starved versus vegetative *D. discoideum* (*n* = 3). FDR, false-discovery rate. **e**, Seahorse analysis of OCR (*n* = 6). **f**, Basal OCR at 8 h (*n* = 6). **g**, OCR due to individual ETC complex activity in isolated mitochondria with saturating substrates and ADP (4 mM). Glutamate (10 mM) and malate (10 mM) were used as substrates for CI, succinate (succ) (10 mM) was used for CII, and ascorbate (asc) (10 mM) with TMPD (100 μM) was used for CIV (*n* = 3). AA, amino acids; rot, rotenone. **h**, OCR responses to mitochondrial perturbations (*n* = 3). FCCP, carbonyl cyanide-*p*-trifluoromethoxyphenylhydrazone; R/A, rotenone and antimycin A. **i**, XTT assay measuring mitochondrial respiration (*n* = 3). *A*_450_, absorbance at 450 nm. **j**, JC-1 staining, indicating Δ*Ψ*_m_ (*n* = 3). **k**, Flow cytometric analysis of MitoTracker Red, which stains actively respiring mitochondria (*n* = 8). MFI, mean fluorescence intensity. **l**, Western blot of OXPHOS complexes (*n* = 4). **m**, Flow cytometric staining of CV subunit ATP5A at 8 h (*n* = 3). **n**, Proliferation of vegetative or starved *D. discoideum* (*n* = 3). **o**, *Dictyostelium discoideum* treated with rotenone or oligomycin from starvation initiation for 8 h (*n* = 3). In **e**, **g**, **h**, data are mean ± s.d. In **f**, **i**, **j**, **k** (right), **m**, **n**, data are mean ± s.e.m. *n* represents independent biological replicates throughout. Statistical significance was calculated using a two-tailed Student’s *t*-test. Source data

**Extended Data Fig. 1:**
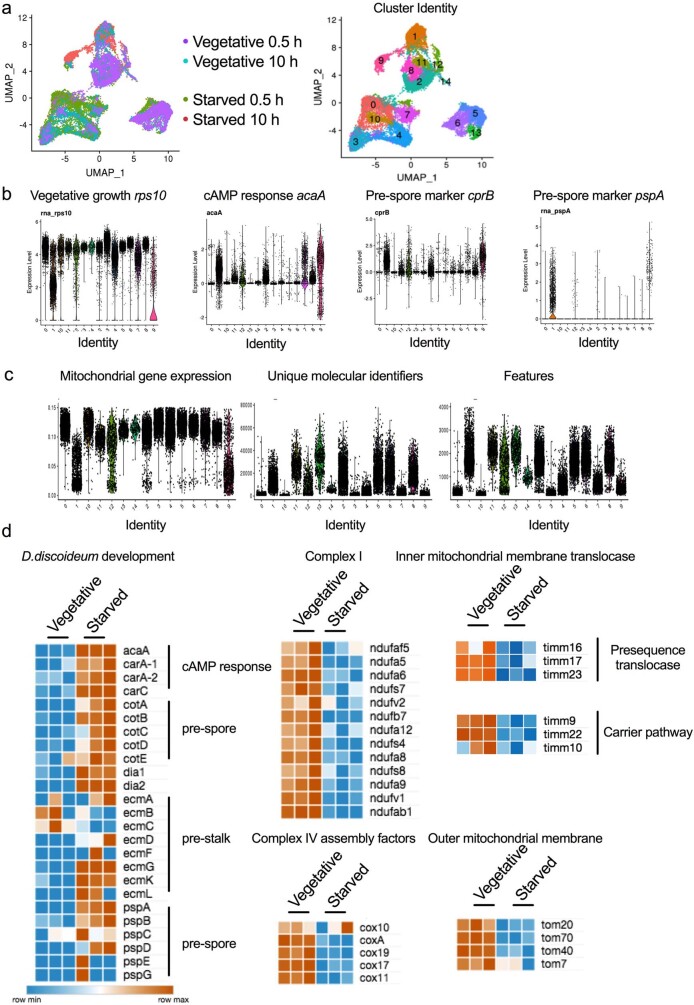
Starving *D. discoideum* decrease expression of genes that encode mitochondrial proteins. **a**, UMAP clustering analysis of single-cell RNA-seq data from vegetative and starved *D. discoideum* cultured for the indicated times demonstrates populations of *D. discoideum* with distinct patterns of gene expression. **b**, Violin plots of established vegetative and developmental marker genes, predictive of the vegetative or developmental state of each cluster. **c**, Violin plots of mitochondrial genes expressed in each cluster, with unique molecular identifiers and feature counts. **d**, Bulk RNA-seq analysis showing increased expression of developmental genes during starvation, with decreased expression of genes for mitochondrial components (including OXPHOS complexes and structural membrane proteins). Single-cell and bulk RNA-seq (**a**–**d**) were performed using three independent biological replicates.

Total nutrient restriction induces aggregation of unicellular *D. discoideum* into a multicellular organism (Fig. 1a), via stages with distinct morphologies (Fig. 1b). Starved *D. discoideum* remodelled their transcriptome (Fig. 1c), and single-cell RNA-sequencing (RNA-seq) revealed discrete populations from 0.5 h of starvation (Extended Data Fig. 1a–c). Metabolic pathways—particularly amino acid metabolism—were highly regulated, which implicates metabolic rewiring in the starvation response (Fig. 1d). Our transcriptomic data agreed well with previous RNA-seq data from *D. discoideum* that were induced to develop by cAMP^14,15^, as starved *D. discoideum* increased expression of genes associated with cAMP signalling, and pre-spore and pre-stalk cells (Extended Data Fig. 1d).

**Extended Data Fig. 2:**
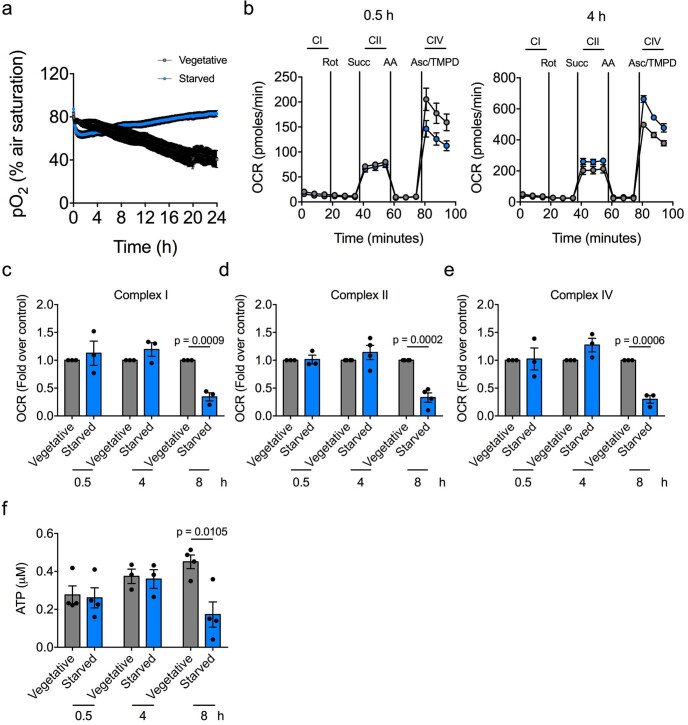
Activity of the ETC complex is intact during early aggregation. **a**, Oxygen tension in the cell culture medium of vegetative *D. discoideum* in HL5 or of starved *D. discoideum* (*n* = 3). **b**, Seahorse analysis of ETC CI, CII and CIV activities in isolated mitochondria provided with saturating substrates at 0.5 and 4 h of starvation. Glutamate (10 mM) and malate (10 mM) were used as substrates for CI, succinate (10 mM) was used as a substrate for CII, and ascorbate (10 mM) with TMPD (100 μM) was used as a substrate for CIV (*n* = 3). **c**–**e**, Fold change in OCR in starved versus vegetative *D. discoideum* at 0.5, 4 and 8 h of culture, owing to the activities of CI (*n* = 3) (**c**), CII (*n* = 4) (**d**) and CIV (*n* = 3) (**e**) in isolated mitochondria provided with saturating substrates. **f**, ATP content in vegetative and starved *D. discoideum* at 0.5, 4 and 8 h (*n* = 4 at 0.5 and 8 h, *n* = 3 at 4 h). In **a**, **b**, data are mean ± s.d. In **c**–**f**, data are mean ± s.e.m. *n* represents independent biological replicates. Statistical significance was calculated using a two-tailed Student’s *t*-test. Source data

Starved *D. discoideum* decreased mitochondrial respiration, reduced their oxygen consumption rate (OCR) and maintained this lower rate, compared to vegetative, nutrient-replete cells (Fig. 1e, f, Extended Data Fig. 2a). The initial decrease in respiration was not due to defective function of electron transport chain (ETC) complexes, as the OCR was similar between mitochondria from vegetative and starved cells when individual complexes were provided with saturating substrates, after up to 4 h of starvation (Extended Data Fig. 2b–e). Prolonged starvation compromised the activities of mitochondrial respiratory chain subunits I, II and IV (CI, CII and CIV, respectively) (Fig. 1g, Extended Data Fig. 2c–e). At advanced starvation, most oxygen consumption was nonmitochondrial: amoebae barely responded to mitochondrial drugs (Fig. 1h) and had a decreased ability to reduce 2,3-*bis*-(2-methoxy-4-nitro-5-sulfophenyl)-2*H*-tetrazolium-5-carboxanilide (XTT) (Fig. 1i), which indicates dampened mitochondrial metabolism. Mitochondrial membrane potential (Δ*Ψ*_m_) rapidly increased initially, and then decreased (Fig. 1j), consistent with lower activity of ETC complexes. MitoTracker Red staining also decreased after 10 h (Fig. 1k). Starving *D. discoideum* specifically decreased ATP synthase (CV) of the oxidative phosphorylation (OXPHOS) machinery, and left CII and CIII unaffected (Fig. 1l, m). Total intracellular ATP declined after 8 h (Extended Data Fig. 2f). These findings may reflect mitochondrial remodelling into a prespore-specific vacuole, which forms the cell wall of the spore^16^.

**Extended Data Fig. 3:**
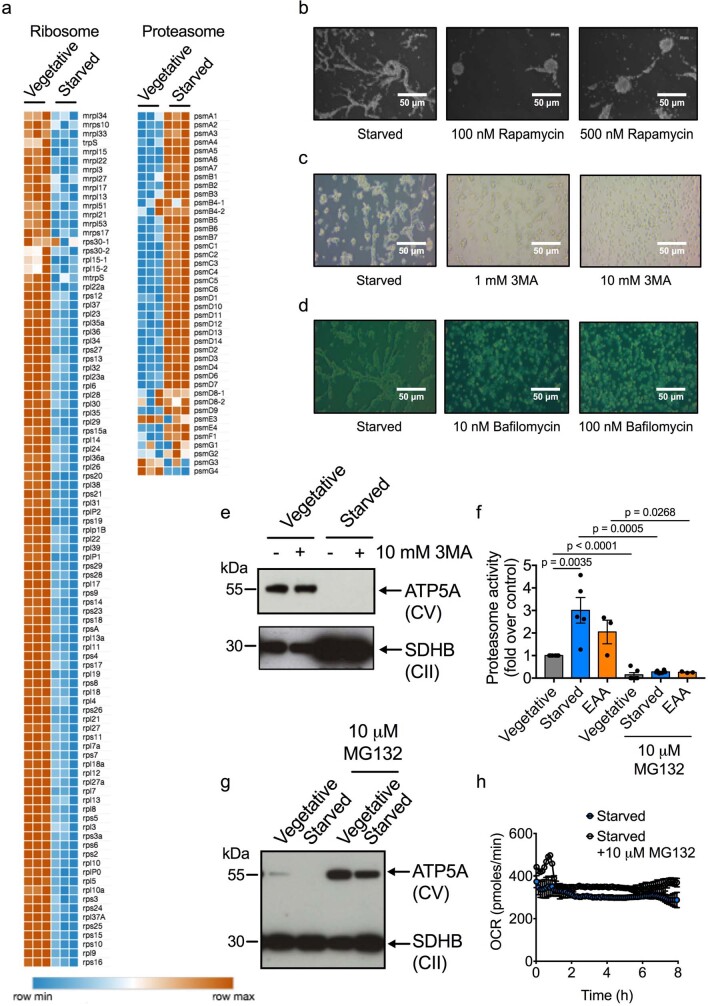
Starving *Dictyostelium* decrease anabolic and increase catabolic processes. **a**, Bulk RNA-seq analysis of ribosomal and proteasomal genes in vegetative and starved *D. discoideum* (*n* = 3). **b**–**d**, *Dictyostelium discoideum* cultured with rapamycin (*n* = 6) (**b**), 3-methyladenine (3MA) (*n* = 6) (**c**) or bafilomycin (*n* = 3) (**d**) from starvation initiation. **e**, Western blot of OXPHOS complexes in vegetative or starved *D. discoideum*, with or without 3MA (*n* = 3). **f**, Relative proteasome activity in vegetative, starved or EAA-supplemented *D. discoideum*, with or without MG132 (*n* = 3). **g**, Western blot of OXPHOS complexes in vegetative or starved *D. discoideum*, with or without MG132 (*n* = 3). **h**, Seahorse analysis of starved *D. discoideum*, with or without MG132 (*n* = 3). In **f**, data are mean ± s.e.m. In **h**, data are mean ± s.d. *n* represents independent biological replicates. Statistical significance was calculated using a two-tailed Student’s *t*-test. Source data

Supporting a role for autophagy (a well-described starvation response^17^) in starving *D. discoideum*, ribosomal genes decreased after 8 h (Extended Data Fig. 3a) as was previously observed^14^. Driving autophagy using rapamycin accelerated aggregation upon starvation (Extended Data Fig. 3b), and inhibition of autophagy blocked aggregation but not degradation of CV (Extended Data Fig. 3c–e). The activity of the 26S proteasome increased (Extended Data Fig. 3f) and degraded CV, as shown by the fact that MG132 inhibition of proteasomal activity preserved CV (Extended Data Fig. 3g). The cytosolic 26S proteasome has also been shown to degrade the intramitochondrial protein UCP2 in mammalian cells^18^. Proteasome inhibition did not restore OCR to rates in starved cells (Extended Data Fig. 3h), which clarified that CV degradation does not drive decreased respiration but may reinforce dampened mitochondrial activity.

## Decreased respiration drives aggregation

**Extended Data Fig. 4:**
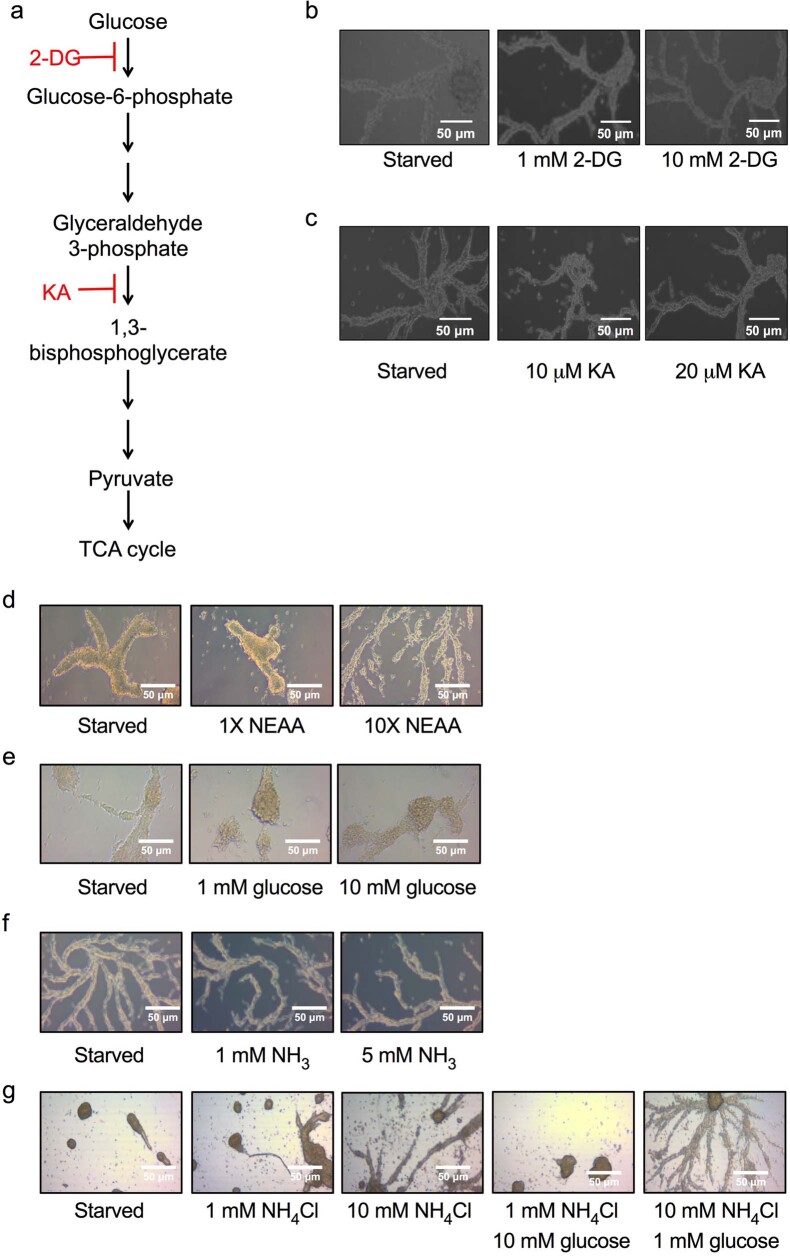
Manipulation of glycolysis, or carbon or nitrogen supply, has no effect on *D. discoideum* aggregation. **a**, Schematic showing targets of the glycolytic inhibitors 2-deoxyglucose (2-DG) and koningic acid (KA). **b**, **c**, Starved *D. discoideum* cultured with 2-DG (*n* = 3) (**b**) or KA (*n* = 3) (**c**). **d**–**g**, Starving *D. discoideum* were cultured with non-essential amino acids (NEAA) (*n* = 3) (**d**), glucose (*n* = 3) (**e**), ammonia NH_3_ (*n* = 3) (**f**) or a combination of glucose and NH_4_Cl (*n* = 3) (**g**) for 8 h.

Starvation halts the proliferation of *D. discoideum* (Fig. 1n), which instead undergo multicellular development. We asked whether the extensive mitochondrial inhibition in starved *Dictyostelium* drives aggregation and multicellularity. Inhibiting CI or CV (Fig. 1o) accelerated aggregation, which indicated that decreased mitochondrial metabolism underlies this response. This aggregation is independent of glycolysis, as it was unaffected by the glycolysis inhibitors 2-deoxyglucose or koningic acid (Extended Data Fig. 4a–c).

## Amino acids rescue mitochondrial changes

**Fig. 2:**
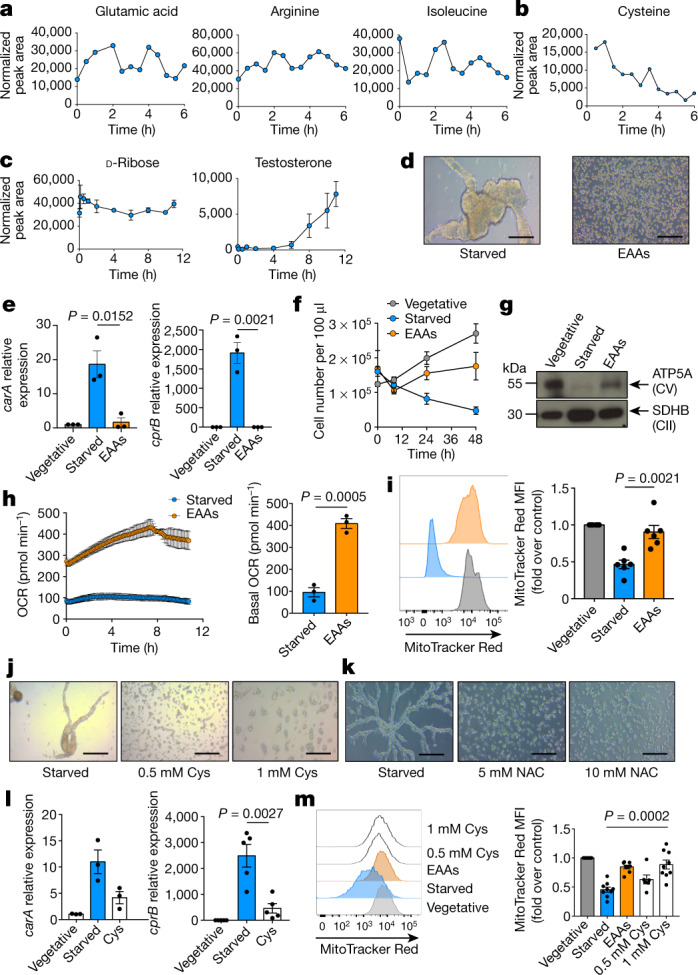
Replacement of amino acids, and of cysteine in particular, rescues aggregation of starving *D. discoideum*. **a**–**c**, Liquid chromatography with mass spectrometry (LC–MS) (**a**, **c**) or gas chromatography with mass spectrometry (GC–MS) (**b**) analysis of starving *D. discoideum* (*n* = 3). **d**, Starving *D. discoideum* cultured with EAAs from starvation initiation (*n* = 20). Scale bars, 50 μm. **e**, mRNA expression of developmental genes *carA* and *cprB* (*n* = 3). **f**, Proliferation of vegetative, starved or EAA-supplemented *D. discoideum* (*n* = 3). **g**, Western blot of OXPHOS complexes (*n* = 3). **h**, OCR in starving *D. discoideum* with or without EAAs (*n* = 3). **i**, MitoTracker Red staining in vegetative, starved or EAA-supplemented *D. discoideum* (*n* = 6). **j**, **k**, Starved *D. discoideum* with l-cysteine (*n* = 16) (**j**) or *N*-acetyl-cysteine (NAC) (*n* = 3) (**k**). **l**, *carA* (*n* = 3) and *cprB* (*n* = 5) mRNA expression. **m**, MitoTracker Red staining of starved *D. discoideum* with l-cysteine (*n* = 9). In **c**, **e**, **f**, **h** (right), **i** (right), **m** (right), data are mean ± s.e.m. In **h** (left), data are mean ± s.d. Statistical significance was calculated using a two-tailed Student’s *t*-test. Source data

We investigated which metabolites were important for this starvation response. Certain amino acids cycled in waves with 2-h periods (Fig. 2a), which is quicker than the population doubling time (Fig. 1n). Cysteine did not cycle but was instead consumed throughout early aggregation, and decreased over time (Fig. 2b). Sugars and steroids remained constant, or increased (Fig. 2c). A variety of systems—including yeast^19,20^ and skeletal muscle^21^—exhibit metabolic oscillations, but the specific, rapid, dynamic regulation of amino acids during *D. discoideum* starvation indicated that they have a special role in this process. Provision of an essential amino acid (EAA) mixture completely reversed aggregation induced by starvation (Fig. 2d), whereas non-essential amino acids delayed aggregation (Extended Data Fig. 4d). This was not due to carbon or nitrogen restoration, as neither glucose nor ammonia (alone or in combination) inhibited aggregation (Extended Data Fig. 4e–g). Previous work has similarly shown that amino acid starvation initiates *D. discoideum* development, and that EAAs inhibit aggregation^22^. EAAs antagonized expression of *carA* and *cprB* (Fig. 2e) (which are markers of starvation-induced cAMP signalling and spore formation, respectively^23^) and overcame the proliferative (Fig. 2f) and mitochondrial defects that accompany starvation, leading to increased CV (Fig. 2g), OCR (Fig. 2h) and MitoTracker Red staining (Fig. 2i).

## Cysteine opposes aggregation

**Extended Data Fig. 5:**
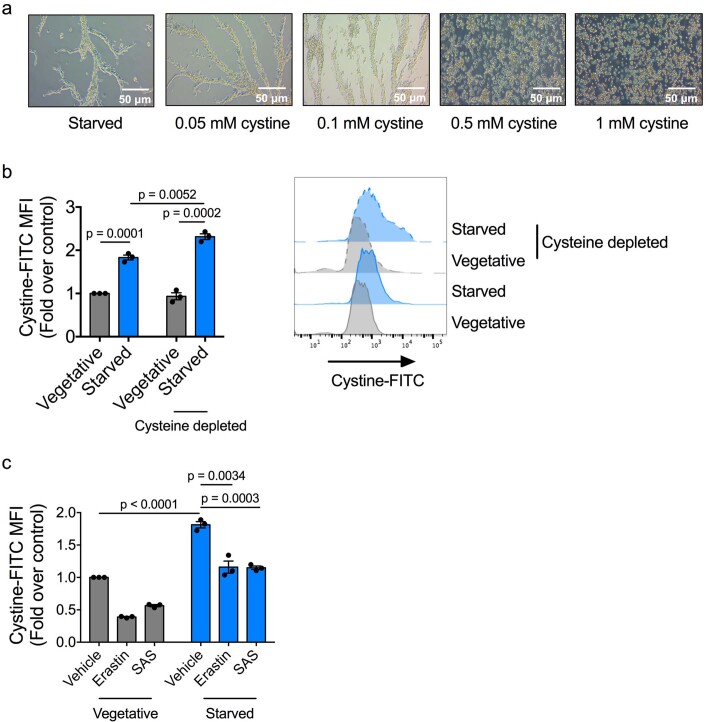
Starving *D. discoideum* take up cystine. **a**, Bright-field images of *D. discoideum* starved for 8 h, with 0.05–1 mM cysteine from starvation initiation (*n* = 3). **b**, Flow cytometric analysis of cystine–FITC staining in vegetative or starved *D. discoideum* that had been maintained in full culture medium or grown in cysteine-depleted medium for 3 h before initiation of starvation (*n* = 3). **c**, Flow cytometric analysis of cystine–FITC staining in vegetative or starved *D. discoideum* that had been cultured in LoFlo medium containing erastin or sulfasalazine (SAS) overnight before initiation of starvation (*n* = 3). In **b**, **c**, data are mean ± s.e.m. *n* represents independent biological replicates. Statistical significance was calculated using a two-tailed Student’s *t*-test. Source data

Although no single amino acid inhibited aggregation completely, only cysteine delayed aggregation (Fig. 2j). *N*-Acetyl-cysteine, a membrane-permeable form of cysteine, completely abrogated aggregation induced by starvation (Fig. 2k), possibly because it was assimilated more quickly than other forms of cysteine. Cysteine blocked starvation-induced expression of *carA* and *cprB* (Fig. 2l), and restored MitoTracker Red staining (Fig. 2m). Starving *D. discoideum* specifically require cysteine. Cystine (two cysteine molecules linked by a disulfide bridge) also antagonized aggregation (Extended Data Fig. 5a). Starved *D. discoideum* took up more of a cystine–fluorescein isothiocyanate (FITC) conjugate than did vegetative cells (Extended Data Fig. 5b). We also cultured cells in cysteine-depleted vegetative medium for 3 h before starvation, to reduce competition for uptake between cystine–FITC and unconjugated cysteine in vegetative medium. Cysteine-depleted and cysteine-replete vegetative cells exhibited similar levels of  cystine–FITC uptake, which indicates that—even after depletion—vegetative cells have no extra cysteine demand. Cysteine-depleted starved cells took up even more cystine–FITC than did starved cells that had not been depleted of cysteine (Extended Data Fig. 5b), which indicates a specific cysteine requirement during starvation. The xCT (also known as Slc7a11) cystine-glutamate transporter mediated at least some of this acquisition, as two xCT inhibitors reduced uptake of cystine–FITC (Extended Data Fig. 5c).

## Amino acids are used for GSH

**Fig. 3:**
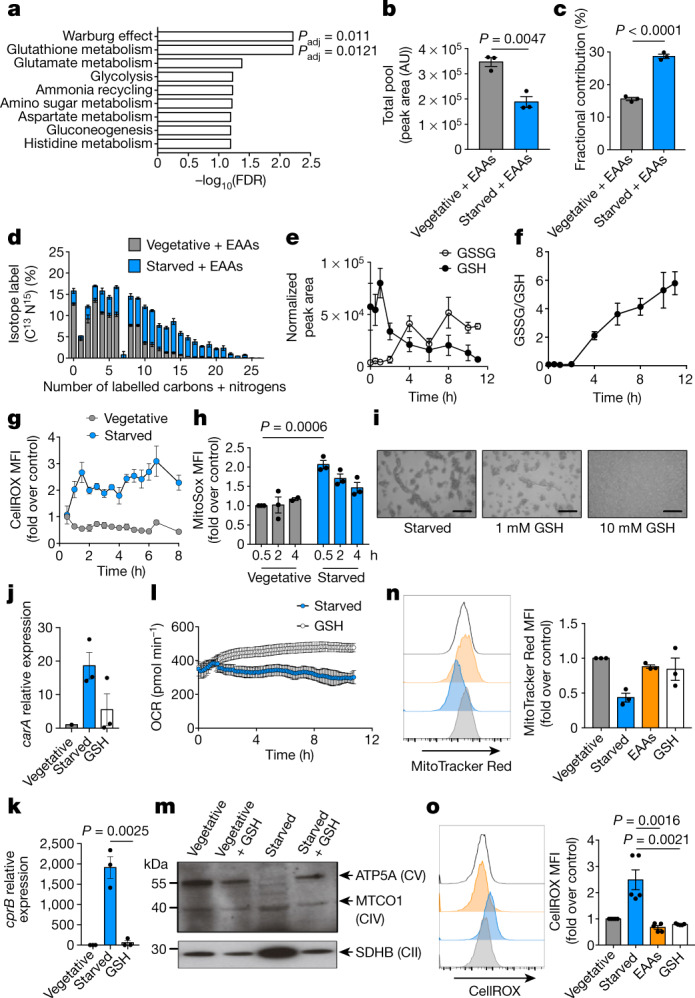
Starving *D. discoideum* use essential amino acids to fulfil increased demand for GSH. **a**, Small Molecule Pathway Database over-representation analysis, using MetaboAnalyst 4.0, of ^13^C^15^N-EAA incorporation in starved *D. discoideum* (*n* = 3). Adjusted *P* value (*P*_adj_) was calculated using a hypergeometric test, followed by the Holm–Bonferroni method. **b**, Total pool size of GSSG in vegetative or starved *D. discoideum* with ^13^C^15^N-EAAs (*n* = 3). AU, arbitrary units. **c**, **d**, Percentage fractional contribution (**c**) or percentage isotope label (**d**) of ^13^C^15^N-EAAs to GSSG (*n* = 3). **e**, **f**, GC–MS analysis of GSSG and GSH during starvation (*n* = 3). **g**, CellROX staining, measuring ROS (*n* = 3). **h**, MitoSox staining, measuring mitochondrial ROS (*n* = 3). **i**, Starving *D. discoideum* with GSH (*n* = 16). **j**, **k**, *carA* (**j**) and *cprB* (**k**) expression in GSH-supplemented cells (*n* = 3). **l**, OCR in starved *D. discoideum* with 10 mM GSH (*n* = 3). **m**, Western blot of OXPHOS complexes in vegetative or starved *D. discoideum* with GSH (10 mM) (*n* = 3). **n**, MitoTracker Red staining of starved *D. discoideum* with EAAs or GSH (10 mM) (*n* = 3). **o**, CellROX staining of cellular ROS in vegetative, starved, EAA-supplemented and GSH-supplemented *D. discoideum* (*n* = 5). In **l**, data are mean ± s.d. In **b**–**h**, **j**, **k**, **n** (right), **o** (right), data are mean ± s.e.m. Statistical significance was calculated using a two-tailed Student’s *t*-test. Source data

**Extended Data Fig. 6:**
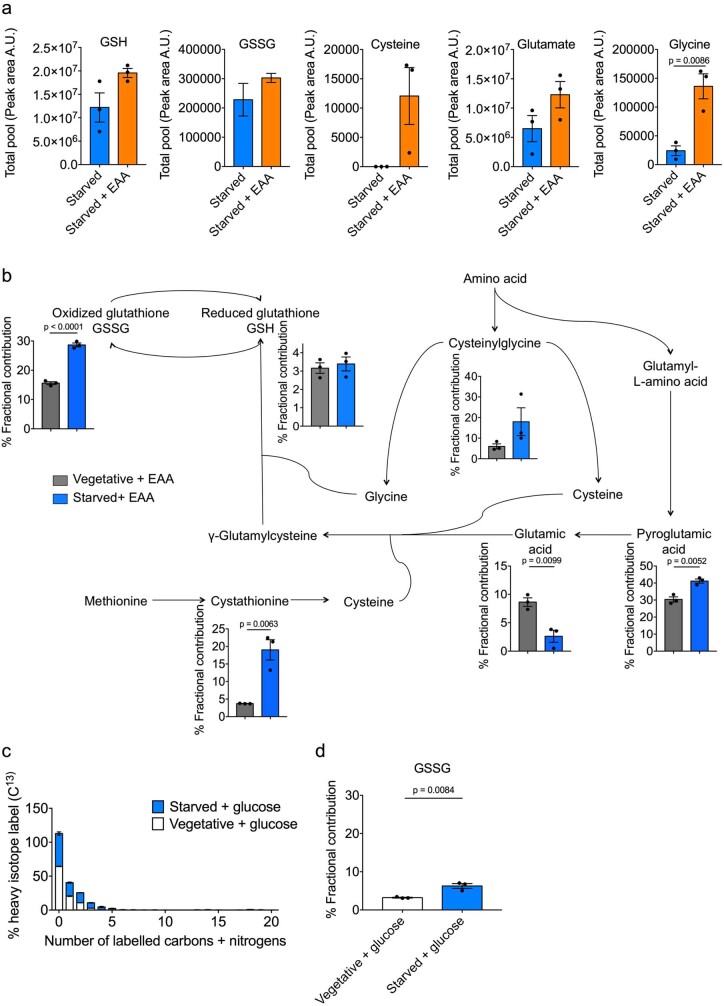
^13^C^15^N-labelled EAAs are used for GSH synthesis in starving *D. discoideum*. **a**, Total pools of GSH, GSSG, cysteine, glutamate and glycine in starved cells with or without EAA supplementation (*n* = 3). **b**, LC–MS metabolite tracing analysis of ^13^C and ^15^N incorporation from ^13^C^15^N-labelled EAAs into vegetative or starved *D. discoideum* (*n* = 3). The schematic shows incorporation of ^13^C and ^15^N into intermediate metabolites of GSH from amino acids. **c**, LC–MS metabolite tracing analysis of ^13^C-glucose into GSSG (*n* = 3). **d**, Percentage fractional contribution of ^13^C-glucose to GSSG labelling in vegetative and starved *D. discoideum* (*n* = 3). In **a**–**d**, data are mean ± s.e.m. *n* represents independent biological replicates. Statistical significance was calculated using a two-tailed Student’s *t*-test. Source data

We traced ^13^C^15^N-labelled EAAs or ^13^C-glucose into starving *D. discoideum* to investigate how EAAs oppose aggregation. Pathways using labelled EAAs (but not glucose) are probably important in this process, as EAAs rescue aggregation whereas glucose does not. Only two pathways—Warburg metabolism and GSH metabolism—were significantly enriched in terms of the number of metabolites that incorporated EAA-derived ^13^C and ^15^N in starved cells (Fig. 3a). Although vegetative cells had more total oxidized glutathione (GSSG) when supplied with ^13^C^15^N-EAAs (Fig. 3b), a greater proportion of GSSG came from labelled EAAs in starved cells (Fig. 3c). Starved cells also incorporated EAA-derived ^13^C and ^15^N into GSSG to a greater extent than did vegetative cells (Fig. 3d). Together, this suggests a critical role for GSH in starvation. GSH and GSSG increased in starved cells that were given unlabelled EAAs, as did the GSH precursors cysteine, glutamate and glycine (Extended Data Fig. 6a), which further demonstrates the use of EAAs in GSH synthesis. Several EAA-derived metabolites that contribute to GSH or cysteine synthesis were differentially labelled in starved versus vegetative cells, indicating altered activity of these pathways (Extended Data Fig. 6b). ^13^C from glucose was not incorporated into GSSG to any great extent in starving or vegetative cells (Extended Data Fig. 6c, d).

## ROS increases GSH demand

**Extended Data Fig. 7:**
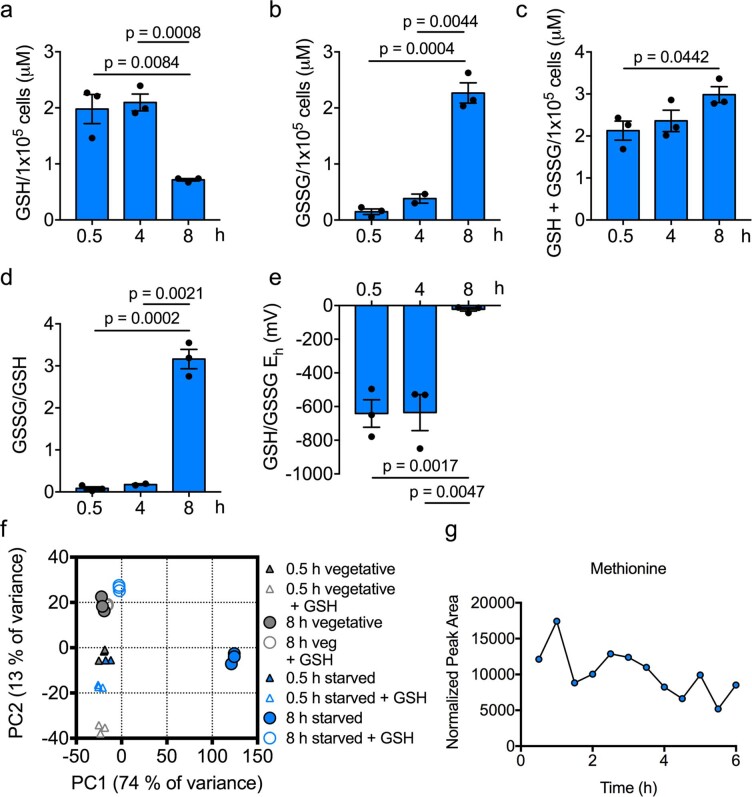
GSH oxidation is increased in starving *D. discoideum*. **a**–**e**, Quantification of GSH (**a**), GSSG (**b**) and total glutathione (**c**) levels, the GSSG/GSH ratio (**d**) and the redox potential (*E*_h_) of the GSH–GSSG couple (**e**) over time in starving *D. discoideum* (*n* = 3). **f**, Principal component analysis of bulk RNA-seq of vegetative or starved *D. discoideum* cultured for 0.5 or 8 h, with or without 10 mM GSH (*n* = 3). **g**, GC–MS analysis of methionine levels in starving *D. discoideum* (*n* = 3). In **a**–**e**, data are mean ± s.e.m. *n* represents independent biological replicates. Statistical significance was calculated using a two-tailed Student’s *t*-test. Source data

Starving *D. discoideum* decreased reduced GSH and raised GSSG (Fig. 3e, Extended Data Fig. 7a, b), which increased total glutathione (Extended Data Fig. 7c). Starvation also increased the GSSG/GSH ratio (Fig. 3f, Extended Data Fig. 7d) and GSH/GSSG oxidation (Extended Data Fig. 7e), which indicates an imbalanced redox state. Supporting previous findings that ROS may be an early pro-aggregation signal^4^, starving *D. discoideum* increased cellular and mitochondrial ROS (mitoROS) (Fig. 3g, h) within 0.5 h. As a consequence, GSH synthesis and oxidation increased, to detoxify ROS.

**Extended Data Fig. 8:**
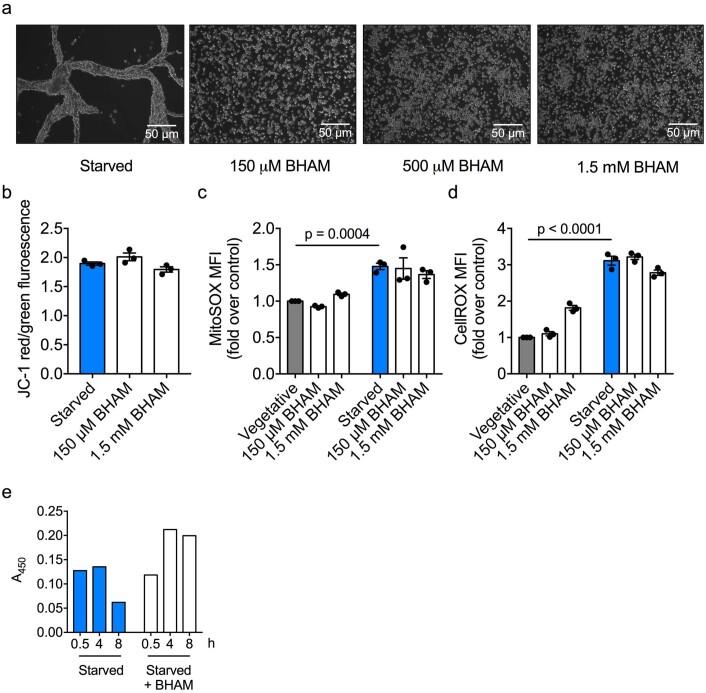
AOX inhibition antagonizes aggregation independently of ROS. **a**, Bright-field images of starved *D. discoideum* cultured for 8 h, with or without BHAM (150 μM–1.5 mM) (*n* = 3). **b**, JC-1 staining indicating Δ*Ψ*_m_ in starved *D. discoideum* cultured for 0.5 h with or without BHAM (*n* = 3). **c**, **d**, MitoSOX (**c**) and CellROX (**d**) staining at 0.5 h (**c**) or 2 h (**d**) of vegetative or starved *D. discoideum*, with or without BHAM (*n* = 3). **e**, XTT assay measuring mitochondrial respiration (*n* = 2). In **b**–**e**, data are mean ± s.e.m. *n* represents independent biological replicates. Statistical significance was calculated using a two-tailed Student’s *t*-test. Source data

Given the increased Δ*Ψ*_m_ and mitoROS, we investigated alternative oxidase (AOX) in aggregation induced by starvation. AOX diverts electrons from the CoQ pool, which limits electron transport to CIII and thereby decreases Δ*Ψ*_m_, mitoROS and ATP synthesis. Inhibiting AOX with benzohydroxamic acid (BHAM) blocked aggregation induced by starvation (Extended Data Fig. 8a). This pro-aggregation effect of AOX is unlikely to be due to Δ*Ψ*_m_ modulation, because BHAM did not affect Δ*Ψ*_m_, mitoROS or cellular ROS (Extended Data Fig. 8b–d). Instead, inhibiting AOX may increase electron flux to CIII, which maintains ETC activity and antagonizes aggregation. Indeed, BHAM increased mitochondrial activity in starved *D. discoideum*, as shown by XTT reduction (Extended Data Fig. 8e).

## GSH mimics EAA supplementation

If starving *D. discoideum* use EAAs for GSH production, the addition of GSH should mimic supplementation with EAAs. As with EAAs, GSH reversed starvation-induced aggregation, mitochondrial defects and transcriptomic changes in *Dictyostelium* (Extended Data Fig. 7f). GSH maintained unicellularity (Fig. 3i), abolished developmental gene expression (Fig. 3j, k), and restored OCR (Fig. 3l), CV (Fig. 3m) and MitoTracker Red staining (Fig. 3n). These data indicate that EAA limitation initiates ROS production, which then promotes aggregation. EAAs and GSH both abolish these ROS (Fig. 3o).

## Cysteine is prioritized for GSH

Cysteine can become conditionally essential in nutrient-restricted contexts^24,25^. In a highly oxidative, starved setting, cysteine may be prioritized for GSH synthesis, which limits its use for other processes. Excess cysteine may oppose *D. discoideum* aggregation by restoring cysteine metabolism beyond GSH synthesis. Uniquely among amino acids, cysteine supplies sulfur for FeS-cluster synthesis, vitamin synthesis, molybdenum cofactor synthesis and transfer RNA (tRNA) thiolation^26–28^. Methionine (the other amino acid that contains sulfur) must first be metabolized to cysteine through several steps to contribute sulfur to these processes^29^. Methionine decreased during starvation (Extended Data Fig. 7g), but did not oppose aggregation—perhaps because its *trans*-sulfuration to cysteine is too slow, or because it is prioritized for other pathways^30^.

FeS clusters are critical functional groups in metabolic enzymes^31^, and their chemical versatility may have supported early life^32^. FeS clusters enable electron transfer by ETC proteins^33^, and their disruption causes mitochondrial dysfunction, metabolic reprogramming^31^ and pathology^33^. tRNA thiolation facilitates translation^27^, which drives proliferation, and new proteins incorporate cysteine itself. Thus, limited cysteine and sulfur metabolism has marked functional consequences.

**Fig. 4:**
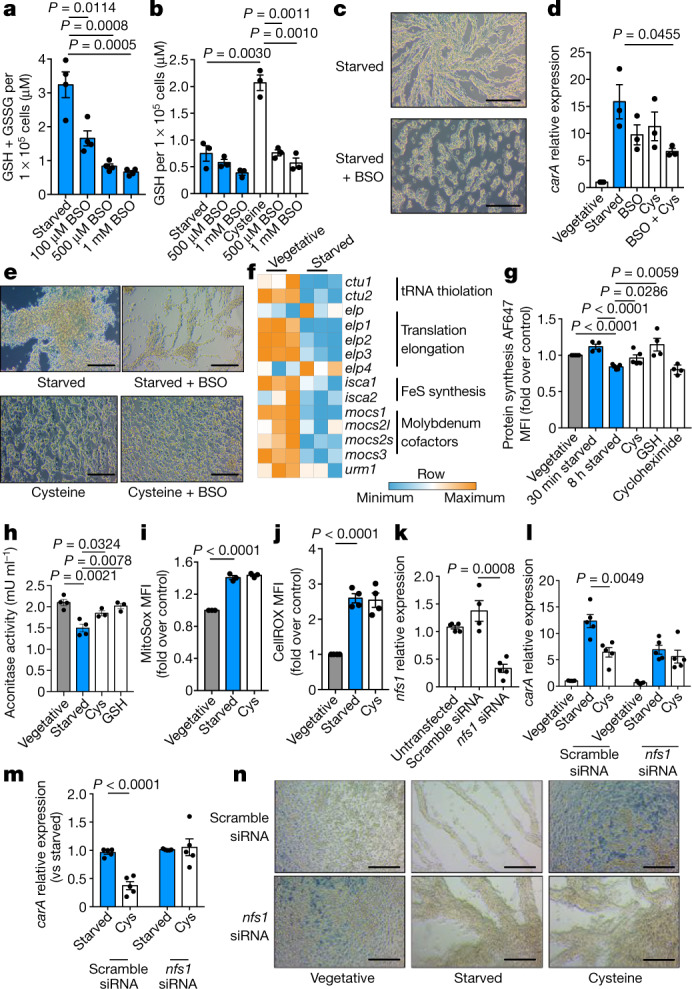
Cysteine delays *D. discoideum* aggregation by restoring sulfur metabolism. **a**, Quantification of GSH and GSSG in starved cells, with or without BSO (*n* = 4). **b**, GSH quantification in starved or cysteine-supplemented cells, with or without BSO (*n* = 3). **c**, Cells starved, with or without BSO (1 mM), for 8 h (*n* = 6). **d**, *carA* expression in starved or cysteine-supplemented cells, with or without BSO (1 mM), at 4 h (*n* = 3). **e**, Starved or cysteine-supplemented cells, with or without BSO, at 8 h (*n* = 6). **f**, RNA-seq of genes for proteins dependent on sulfur from cysteine (*n* = 3). **g**, Protein synthesis in *D. discoideum* after 8-h vegetative, 30-min or 8-h starved, or 8-h cysteine- or GSH-supplemented culture (*n* = 4). As a negative control for protein synthesis, cells were treated with cycloheximide for 1 h. **h**, Mitochondrial aconitase activity in *D. discoideum* after 8-h vegetative, starved or Cys- or GSH-supplemented culture (*n* = 4). **i**, **j**, MitoSOX (*n* = 3) (**i**) and CellROX (*n* = 4) (**j**) staining of vegetative, starved or cysteine-supplemented *D. discoideum*. **k**, *nfs1* expression (*n* = 4). **l**, **m**, *carA* expression, relative to vegetative cells with scramble siRNA (**l**), or to the starved condition for each siRNA (**m**) (*n* = 5). **n**, Vegetative, starved or cysteine-supplemented *D. discoideum* with scramble or *nfs1*-targeting siRNA (*n* = 6). In **a**, **b**, **d**, **g**–**m**, data are mean ± s.e.m. Statistical significance was calculated using a two-tailed Student’s *t*-test. Scale bars, 50 μm (**c**, **e**, **n**). Source data

**Extended Data Fig. 9:**
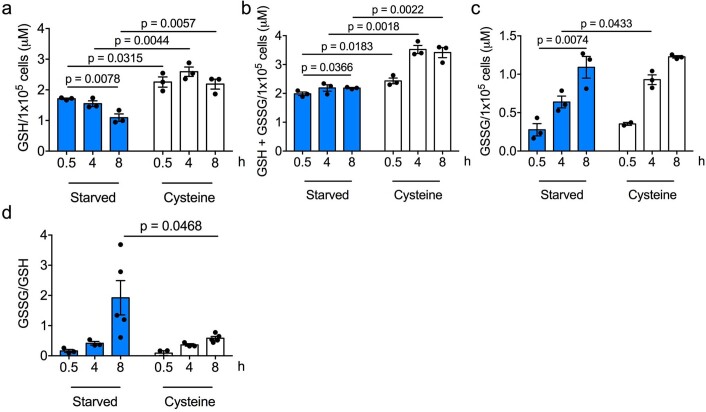
Cysteine is used to make GSH in starved *D. discoideum*. **a**–**d**, Quantification of GSH (**a**), total glutathione (**b**) and GSSG (**c**) levels, and the GSSG/GSH ratio (**d**) over time in starving *D. discoideum*, with or without cysteine supplementation (*n* = 3 independent biological replicates). Data are mean ± s.e.m. Statistical significance was calculated using a two-tailed Student’s *t*-test. Source data

To investigate whether cysteine is funnelled into GSH upon starvation, we examined GSH in starved *D. discoideum* supplemented with cysteine, with and without buthionine sulfoximine (BSO). BSO inhibits γ-glutamyl synthetase (γGCS), which conjugates cysteine to glutamate in the first step of GSH synthesis. BSO decreased GSH (Fig. 4a), confirming inhibition of GSH synthesis. Cysteine increased GSH and total glutathione (Extended Data Fig. 9a, b) after 30 min of starvation; these effects were maintained after up to 8 h of starvation, indicating that starving *D. discoideum* use cysteine for GSH. Cysteine did not greatly affect GSSG content (Extended Data Fig. 9c), but reversed the starvation-induced increase in the GSSG/GSH ratio (Extended Data Fig. 9d), probably because cysteine increased GSH. BSO blocked this ability of cysteine to increase GSH (Fig. 4b), which shows that starving *D. discoideum* direct cysteine into GSH using γGCS. BSO alone inhibited aggregation (Fig. 4c–e), possibly because it preserved endogenous cysteine to support sulfur- and cysteine-dependent processes other than GSH synthesis. BSO and cysteine additively blocked multicellular development, as the combination decreased *carA* expression (Fig. 4d) and delayed aggregation (Fig. 4e) more than did either agent alone. These results suggest that cysteine opposes *D. discoideum* aggregation not by supporting GSH synthesis, but instead by restoring other cysteine-dependent processes in starving cells.

## Starvation limits sulfur metabolism

**Extended Data Fig. 10:**
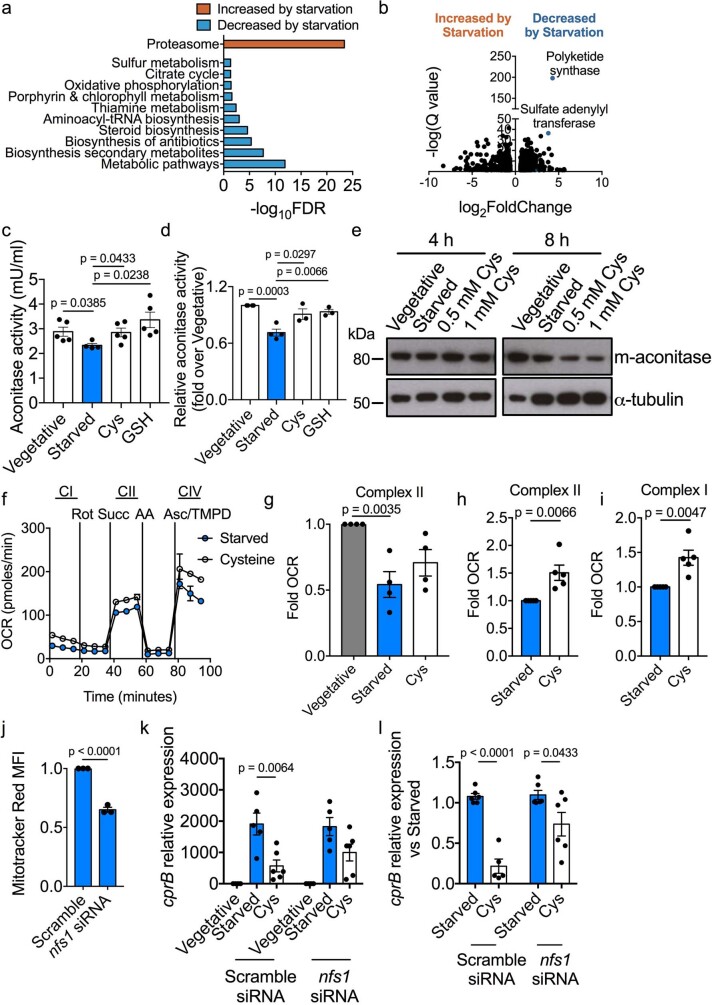
Cysteine restores sulfur-dependent processes in starving *D. discoideum*. **a**, KEGG pathway analysis of proteomics data, showing significantly regulated pathways in starved versus vegetative *D. discoideum* cultured for 8 h (*n* = 3). **b**, Volcano plot of proteomics analysis of starved compared to vegetative *D. discoideum* at 8 h (*n* = 3). **c**, Mitochondrial aconitase activity in *D. discoideum* after 4-h vegetative, starved or cysteine- or GSH-supplemented culture (*n* = 5). **d**, Fold change in mitochondrial aconitase activity in *D. discoideum* after 8-h vegetative, starved or cysteine- or GSH-supplemented culture (*n* = 3). **e**, Western blot of mitochondrial aconitase levels after 4 and 8 h (*n* = 3). **f**–**i**, Seahorse analysis of ETC CI, CII and CIV activities in mitochondria isolated from starved or cysteine-supplemented *D. discoideum* after 8 h, and provided with saturating substrates (*n =* 4). Glutamate (10 mM) and malate (10 mM) were used as substrates for CI, succinate (10 mM) was used as a substrate for CII, and ascorbate (10 mM) with TMPD (100 μM) was used as a substrate for CIV. **j**, MitoTracker Red staining of starved *D. discoideum* transfected with scramble siRNA or siRNA targeting *nfs1* (*n* = 3). **k**, **l**, mRNA expression of *cprB*, relative to vegetative cells transfected with scramble siRNA (**k**) or relative to the starved condition for each siRNA (**l**) (*n* = 5). In **c**, **d**, **g**–**l**, data are mean ± s.e.m. In **f**, data are mean ± s.d. *n* represents independent biological replicates. Statistical significance was calculated using a two-tailed Student’s *t*-test. Source data

We next investigated whether sulfur limitation was the signal that drove multicellular development, and whether cysteine antagonized development by supplying sulfur. We hypothesized that if starving *D. discoideum* pull cysteine into GSH synthesis, other sulfur-dependent processes should consequently decrease. Starved *D. discoideum* decreased their expression of genes involved in cysteine-derived sulfur metabolism, including tRNA thiolation (*ctu1* and *ctu2*), molybdenum cofactor production (*mocs1*, *mocs2l*, *mocs2s* and *mocs3*) and FeS-cluster synthesis (*isca1* and *isca2*) (Fig. 4f). Proteomics analysis revealed that sulfur metabolism was significantly decreased in starving *D. discoideum* (Extended Data Fig. 10a), with sulfate adenylyl transferase (which is involved in sulfur assimilation^23^) being one of the most decreased proteins (Extended Data Fig. 10b, Supplementary Table 1). The only significantly increased pathway was proteasome-related (Extended Data Fig. 10a), consistent with increased proteasome activity upon starvation (Extended Data Fig. 3f) and with previous studies in cAMP-pulsed *D. discoideum*^34^.

We then examined a range of sulfur-dependent processes. Protein synthesis incorporates cysteine into new proteins, proteins of the translation elongator complex are sulfur-dependent^35^ and tRNA thiolation facilitates translation^27^. Starvation caused an initial and transient increase in protein synthesis, possibly to produce proteins for motility, aggregation and the differentiation of stalk and spore cells (Fig. 4g). After 8 h, protein synthesis dropped to levels similar to those observed with the protein synthesis inhibitor cycloheximide. This may result from decreased tRNA thiolation or activity of proteins of the translation elongator complex (which are reduced at the mRNA level (Fig. 4f)), and from a reduced availability of cysteine for protein synthesis. Cysteine and GSH restored translation in 8-h starved cells (Fig. 4g): GSH may further restore translation by providing cysteine, glutamate and glycine, in addition to its antioxidant effects.

Enzymes containing FeS clusters depend on sulfur for their activity. For example, aconitase needs its FeS cluster to metabolize citrate to isocitrate in the tricarboxylic acid (TCA) cycle. *Dictyostelium discoideum* decreased the activity of mitochondrial aconitase after 4 h of starvation (Extended Data Fig. 10c), which was even more pronounced after 8 h (Fig. 4h, Extended Data Fig. 10d). It is the enzyme activity of aconitase that is regulated (at least after 4 h), as absolute levels of the enzyme are unchanged (Extended Data Fig. 10e). Supporting this, although levels of mitochondrial aconitase protein decreased after 8 h, cysteine restored enzyme activity and not expression (Fig. 4h, Extended Data Fig. 10e). GSH also restored the activity of mitochondrial aconitase (Fig. 4h). These data indicate that sulfur restriction may modulate the activity of FeS-cluster-containing enzymes. Iron starvation dissociates FeS clusters from mammalian cytosolic aconitase, decreasing its metabolism of citrate to isocitrate but maintaining its levels to perform its alternative activity of mRNA stabilization^36^. Similarly, sulfur limitation may dissociate FeS clusters from FeS-cluster-containing enzymes to decrease activity. To bolster these findings, we examined ETC CII, another FeS-dependent enzyme. Succinate-driven CII activity decreased after 8 h of starvation, and was rescued by cysteine (Extended Data Fig. 10f–h). Cysteine did not restore CII activity to levels in vegetative cells (Extended Data Fig. 10g), but did significantly increase its activity compared to starved cells (Extended Data Fig. 10h)—probably because sulfur from supplemented cysteine is used to recover several sulfur-dependent processes. For example, cysteine also rescued the glutamate- and malate-fuelled activity of CI, another FeS-dependent enzyme (Extended Data Fig. 10i).

Mitochondrial superoxide can degrade the FeS cluster of mitochondrial aconitase. Cysteine, unlike GSH, did not decrease starvation-induced mitochondrial or cellular ROS (Fig. 4i, j). GSH directly detoxifies ROS, whereas cysteine must first be processed to GSH to mediate such potent antioxidant activity. This suggests that cysteine rescues the activity of mitochondrial aconitase by restoring sulfur. These results reinforce the idea that ROS induce GSH and pull cysteine into GSH synthesis. This limits sulfur metabolism to decrease translation and proliferation, thus promoting multicellularity during starvation. Supplemented GSH opposes this both by directly abolishing the initial ROS signal and preserving endogenous cysteine to maintain sulfur-dependent metabolism, explaining the strong reversal of starvation-induced aggregation by GSH supplementation. Cysteine supplementation antagonizes the multicellular development of *D. discoideum* not by reducing ROS, but instead by feeding in downstream of this signal to restore sulfur and rescuing several sulfur metabolic processes (including translation and sulfur-dependent enzyme activity).

## Sulfur metabolism determines cell fate

If cysteine opposes multicellular development by maintaining sulfur metabolism, limiting sulfur liberation from cysteine should abolish the ability of cysteine to rescue aggregation. Nitrogen fixation 1 (NFS1) cysteine desulfurase^26^ removes sulfur from cysteine for processes that include FeS-cluster synthesis^37^ and tRNA thiolation^38^. *nfs1*-targeting small interfering RNA (siRNA) (Fig. 4k) inhibited the ability of cysteine to antagonize aggregation (Fig. 4l–n). Knockdown of *nfs1* decreased MitoTracker Red staining in starved *D. discoideum* (Extended Data Fig. 10j), consistent with accelerated aggregation in these cells. Starvation-induced *carA* expression was lower in *nfs1*-silenced cells compared to control cells (Fig. 4l), and we suggest that this is because they have already passed the peak of *carA* expression due to accelerated development. Cysteine did not decrease starvation-induced expression of *carA* in cells that lack NFS1 (Fig. 4l, m), and was less effective at antagonizing *cprB* expression (Extended Data Fig. 10k, l). This is reflected in the aggregation process. Starved, *nfs1*-silenced *D. discoideum* accelerated aggregation compared to control cells, and cysteine could not antagonize aggregation in *nfs1*-silenced cells (Fig. 4n). These data indicate that sulfur liberation from cysteine by NFS1 is responsible for cysteine antagonism of *D. discoideum* aggregation.

We present a model in which rapid ROS production by starving *D. discoideum* increases demand for the antioxidant GSH. These ROS, which increase during nutrient restriction^39–41^, prioritize cysteine for GSH synthesis, which limits the use of the sulfur from cysteine for other metabolic processes. Such sulfur restriction decreases mitochondrial metabolism and protein synthesis, inhibiting proliferation and promoting aggregation and multicellular development during starvation. Thus, we reveal a mechanism by which a sulfur-dependent metabolic switch dictates cell function. Numerous cell types (notably immune cells and cancer cells) rewire their metabolism to alter function^42^ and sulfur use may be important in these settings, particularly in proliferative cells or in immune cells entering nutrient-restrictive environments.

Some cancer cells preserve sulfur metabolism, with NFS1 being highly expressed in lung adenocarcinoma to maintain FeS clusters to promote cell survival^43^. Cysteine-restricted tumour cells increase methionine *trans*-sulfuration to cysteine to support growth^44^, and starving cancer cells of cysteine or cystine enhances checkpoint-blockade efficacy and antitumour immunity^45^. Although ROS have numerous roles and increase during starvation^39^, research currently focuses on oxidative modifications and redox balance, whereas here we show an entirely different ROS signalling mechanism. MitoROS control haematopoietic stem cell differentiation and proliferation^46^, and are essential for keratinocyte differentiation^47^, and GSH/GSSG status acts as a switch between differentiation and proliferation^48^; however, sulfur metabolism has not been examined in this context. Investigating how metabolic processes influence cell function in early life forms may provide new insights into more complex nutrient utilization pathways in mammalian cells. Our work reveals a ROS signalling mechanism that controls a sulfur-dependent metabolic switch to dictate cell fate and multicellular development.

## Methods

No statistical methods were used to predetermine sample size. The experiments were not randomized, and investigators were not blinded to allocation during experiments and outcome assessment.

### *Dictyostelium* culture

*Dictyostelium discoideum* strain Ax4 was purchased from the Dictybase stock centre. Vegetatively growing cells were axenically maintained in shaking culture in HL5 nutrient medium (14.3 g l^−1^ bacto peptone, 7.15 g l^−1^ yeast extract, 18 g l^−1^ maltose monohydrate, 0.641 g l^−1^ Na_2_HPO_4_, 0.49 g l^−1^ KH_2_PO_4_, supplemented with biotin, cyanocobalamin, folic acid, lipoic acid, riboflavin and thiamine-HCl). Starvation and consequent aggregation were induced by washing *D. discoideum* four times in development buffer (5 mM Na_2_HPO_4_, 5 mM KH_2_PO_4_, 1 mM CaCl_2_, 2 mM MgCl_2_ in autoclaved H_2_O) and plating at a density of 2 × 10^6^ cells per ml in development buffer on tissue-culture-treated plates, without shaking. As a control, vegetatively growing cells were plated at a density of 2 × 10^6^ cells per ml in HL5, or LoFlo for flow cytometric experiments, on tissue-culture-treated plates, without shaking.

### Drug and metabolite treatments

All drugs and supplemented metabolites were added at initiation of starvation, unless otherwise stated. BHAM (150 μM–1.5 mM), oligomycin (10 μM), FCCP (100 nM–5 μM), rotenone (100 nM–1 μM), succinate (10 mM), ascorbate (10 mM), TMPD (100 μM), glutamate (10 mM), malate (10 mM), antimycin A (10 μM), ADP (4 mM), l-cysteine (100 μM–2 mM), cystine, (0.05–1 mM) *N*-acetyl cysteine (0.5–10 mM), reduced GSH (1–20 mM), 2-deoxyglucose (1–10 mM), koningic acid (10–20 μM), glucose (1–20 mM), NH_3_ (1–5 mM), NH_4_Cl (1–10 mM), 3-methyladenine (1–10 mM), MG132 (10 μM), l-buthionine sulfoximine (100 μM–1 mM) and erastin (100 μM) were all from Sigma. Bafilomycin (10–100 nM) was from Cell Signaling. Rapamycin (20–500 nM) was from LC Laboratories. Sulfasalazine (1 mM) was from Tocris Bioscience. MitoParaquat (1–10 μM) was from Abcam. We diluted 10× essential amino acids from 50× MEM amino acids solution (ThermoFisher), for final concentrations of 6 mM l-arginine hydrochloride, 1 mM l-cystine, 2 mM l-histidine hydrochloride-H_2_O, 4 mM l-isoleucine, 4 mM l-leucine, 4 mM l-lysine hydrochloride, 1 mM l-methionine, 2 mM l-phenylalanine, 4 mM l-tryptophan, 2 mM l-tyrosine and 4 mM l-valine.

### Proliferation by cell counting

Two hundred thousand cells per condition were plated in 100 μl HL5 or development buffer in a 96-well tissue-culture-treated plate. At the time of counting, cells and medium were collected and diluted 1:3 with PBS. Ten μl of 123count eBeads counting beads (Thermo Scientific) of known concentration were added to each sample. Three thousand beads were counted per sample, and cell number was calculated according to the manufacturer’s instructions.

### RNA-seq

Total RNA was isolated using the RNEasy kit (Qiagen) and quantified using a Qubit 2.0 (ThermoFisher). Libraries were prepared using the TruSeq stranded mRNA kit (Illumina) and sequenced in a HISeq 3000 (Illumina) by the Deep-sequencing Facility at the Max Planck Institute for Immunobiology and Epigenetics. Sequenced libraries were processed with deepTools^49^, using STAR^50^, for trimming and mapping, and featureCounts^51^ to quantify mapped reads. Reads were mapped to the dicty 2.7 genome assembly. Raw mapped reads were processed in R (Lucent Technologies) with DESeq2^52^ to generate normalized read counts to visualize as heat maps using Morpheus (Broad Institute) and determine differentially expressed genes with greater than 2 fold change and lower than 0.1 adjusted *P* value, which were analysed for pathway enrichment using STRING.

### Single-cell RNA-seq

Single-cell RNA-seq was performed using a 10X Genomics Chromium Controller. Single cells were processed with GemCode Single Cell Platform using GemCode Gel Beads, Chip and Library Kits (v.2) following the manufacturer’s protocol. An estimated 28,000 cells were sequenced from an initial 7,000 cells added. Libraries were sequenced on HiSeq 3000 (Illumina). Samples were demultiplexed and aligned using Cell Ranger 2.2 (10X genomics) to genome build release 2-12, then processed and analysed in R using Seurat v.3 and uniform manifold approximation and projection (UMAP) as a dimensionality reduction approach.

### Seahorse analysis

Two hundred thousand cells per well were plated on a Seahorse XFp 8-well plate in 40 μl HL5 or development buffer and allowed to adhere. Then, 110 μl of the appropriate medium was added to the wells for a final volume of 150 μl. OCR was measured using the Seahorse XFp (Seahorse Bioscience) maintained at 22 °C. The AOX inhibitor BHAM (1.5 mM), CV inhibitor oligomycin (10 μM), mitochondrial membrane ionophore FCCP (5 μM), CI inhibitor rotenone (1 μM) and CIII inhibitor antimycin A were injected as indicated.

### SDR measurement of oxygen tension

*Dictyostelium discoideum* were plated on a 24-well OxoDish OD24 at a density of 2 × 10^6^ cells per ml in either HL5 or development buffer, and oxygen tension in the cell culture medium was measured using the SDR SensorDish Reader (PreSens).

### XTT assay

*Dictyostelium discoideum* were plated at a density of 2 × 10^6^ cells per ml in HL5 and were allowed to adhere for 1 h. The medium was then carefully removed and replaced with development buffer or fresh HL5. At the end of the time course, samples were analysed using the CyQUANT XTT Cell Viability Assay (ThermoFisher) according to the manufacturer’s instructions. Superoxide dismutase (Sigma) was included with each condition to remove superoxide as a confounding factor.

### ATP assay

*Dictyostelium discoideum* were plated at a density of 2 × 10^6^ cells per ml in HL5 and were allowed to adhere for 1 h. The medium was then carefully removed and replaced with development buffer or fresh HL5. At the end of the time course, samples were analysed using the ATP determination kit (Thermo Scientific) according to the manufacturer’s instructions.

### Flow cytometry

*Dictyostelium discoideum* were cultured in shaking culture in low fluorescence axenic LoFlo medium (ForMedium) overnight before the experiment, and were then treated as desired, using LoFlo medium in place of HL5 medium. Two hundred thousand cells per condition were plated in 100 μl LoFlo medium in a 96-well tissue culture-treated plate, and allowed to adhere for 1 h, ensuring consistent adherence between samples. The culture medium was then carefully removed and changed to the medium of interest (development buffer alone or supplemented with indicated nutrients or drugs), or replaced with fresh LoFlo medium, and cells were incubated for the indicated times. Thus, starved cells always had counterpart control cells that had been cultured in a full complement of nutrients for the same time, and these whole populations could be accurately compared. Then, 30 min before the end of the treatment, cells were collected, disaggregated and stained in PBS for 30 min. This was done to diminish any residual autofluorescence in LoFlo medium, to stop any binding of peptone or yeast extract proteins binding to cell stains, and to ensure that differences in flow cytometric results were not due to staining in different base media of different fluorescence. Cells were washed using 1× Perm/Wash Buffer (BD Biosciences) and resuspended in PBS. If a fixation step was needed, cells were fixed for 20 min at 4 °C in Fixation/Permeabilization solution (BD Biosciences), washed using 1× Perm/Wash Buffer and resuspended in PBS. Cells were collected using the Fortessa or LSR II flow cytometers (BD Biosciences), with the software FACSDiva (BD Biosciences). An example gating strategy for identifying live, single vegetative or starved *D. discoideum* cells is shown in Supplementary Fig. 2. Analysis was performed using FlowJo software (TreeStar). Dyes used were MitoTracker Red, Live/Dead Aqua, Live/Dead Blue, Live/Dead Near-IR, MitoSOX and CellROX (all from ThermoFisher Scientific), anti-ATP5A–FITC (abcam), JC-1 (Thermo scientific) and BioTracker Cystine–FITC Live Cell Dye (Merck).

### Western blotting

Two million cells per condition were plated in 1 ml HL5 of the appropriate medium, with or without indicated drugs or nutrients, in a 12-well tissue-culture-treated plate. At the end of the stimulation, supernatant was removed and cells were directly lysed in 1× cell lysis buffer (Cell Signaling) containing 1 mM PMSF. Protein was quantified using a BSA assay. Then, 1× loading dye and 1 mM DTT were added to samples, which were then heated at 95 °C for 5 min. Samples were run on pre-cast 4% to 12% bis-tris protein gels (Life Technologies). Proteins were transferred to nitrocellulose membranes using the iBLOT 2 system (Life Technologies), and blocked with 5% w/v milk and 0.1% v/v Tween-20 in Tris-buffered saline (TBS-T) for 1 h at room temperature. Membranes were incubated with primary antibodies in 5% w/v BSA in TBS-T overnight at 4 °C, washed 3 times with TBS-T, and incubated with the appropriate horseradish-peroxidase-conjugated secondary antibody (Pierce; dilution 1:10,000) in 5% w/v BSA in TBS-T for 1 h at room temperature. After 3 further washes with TBS-T, membranes were incubated for 5 min with SuperSignal West Pico or Femto Chemiluminescent Substrate (Pierce). Bands were visualized on Biomax MR film (Kodak) using a developer. OXPHOS complexes were probed with the Total OXPHOS Rodent WB Antibody Cocktail (Abcam; dilution 1:1,000). The 12G10 anti-α-tubulin-s antibody for *D. discoideum* (dilution 1:1,000) was from the Developmental Studies Hybridoma Bank (DSHB) at the University of Iowa. The mitochondrial aconitase (aconitase 2) antibody was from Abcam (dilution 1:1,000).

### Metabolomic profiling

#### Discovery metabolomics by GC–MS

Discovery metabolomics by GC–MS was carried out using an Agilent 7890 gas chromatograph in-line with an Agilent 5977 single quadrupole mass spectrometer. Dry samples were derivatized with *N*-methyl-*N*-(trimethylsilyl)-trifluoroacetamide. Gas chromatography separation was on a HP-DB5 column (30 mm × 0.25 mm) with a temperature gradient from 80 °C to 320 °C. The mass spectrometer was operated in full scan mode with a mass range of 50 to 500 *m*/*z*. Data processing was performed using an R script developed in-house. Features were annotated by matching of retention times to standard compounds and matching of fragmentation spectra to the Human Metabolomics Database (HMDB).

#### Metabolite quantification by LC–MS

Cells were centrifuged for 2 min at 500*g* at 4 °C. The pellet was washed with ice-cold PBS and centrifuged for 2 min at 500*g* at 4 °C. The supernatant was discarded. Samples were extracted in 750 μl 50:30:20 v/v/v methanol/acetonitrile/water, and samples were centrifuged for 10 min at maximum speed at 4 °C. The supernatant was stored at −80 °C. Targeted metabolite quantification by LC–MS was carried out using an Agilent 1290 Infinity II UHPLC in-line with an Agilent 6495 QQQ-MS operating in multiple reaction monitoring (MRM) mode. MRM settings were optimized separately for all compounds using pure standards. Liquid chromatography separation was on a Phenomenex Luna propylamine column (50 × 2 mm, 3-μm particles) using a solvent gradient of 100% buffer B (5 mM ammonium carbonate in 90% acetonitrile) to 90% buffer A (10 mM NH_4_ in water). Flow rate was from 1,000 to 750 μl min^−1^. Autosampler temperature was 5 °C and injection volume was 2 μl. Data processing was performed by an R script developed in-house.

#### Metabolite tracing analysis

Five million vegetative or starved cells were cultured in the presence or absence of ^13^C^15^N-essential amino acids, unlabelled amino acids, ^13^C-glucose or unlabelled glucose for 6 h. Samples were extracted in 750 μl 50:30:20 v/v/v methanol/acetonitrile/water, as for metabolite quantification. Label tracing by LC–MS was carried out using an Agilent 1290 Infinity II UHPLC in-line with a Bruker impact II QTOF-MS operating in negative ion mode. Scan range was from 20 to 1,000 Da. Mass calibration was performed at the beginning of each run. Liquid chromatography separation was performed as for targeted metabolite quantification. X13CMS software^53^ was used to compare incorporation of stable heavy-isotope-labelled nitrogen or carbon derived from ^15^N, ^13^C amino acids or ^13^C glucose into polar metabolites between starved and non-starved cells. Metabolites with significantly different (*P* < 0.05) total pool sizes or per cent isotope incorporation from either ^13^C or ^15^N were identified by accurate mass using the HMDB. For pathway analysis, metabolites assigned a Kyoto Encyclopedia of Genes and Genomes (KEGG) identifier in the HMDB were searched using the KEGG Mapper-Search Pathway tool. Metabolites of interest from the top 2 most significantly changed pathways were further analysed by targeted analysis, in which metabolites were quantified using AssayR^54^ and identified by matching accurate mass and retention time to standards.

### Quantitative PCR analysis

Total RNA was extracted using the RNeasy mini kit (Qiagen) and quantified using a Qubit 2.0. cDNA was prepared using 20–100 ng μl^−1^ total RNA by a reverse-transcription PCR (RT–PCR) using a High Capacity cDNA Reverse Transcription kit (Applied Biosystems), according to the manufacturer’s instructions. Quantitative PCR was performed on cDNA using SYBR Green probes, on an Applied Biosystems 7000 sequence detection system, using iTaq Universal SYBR Green Supermix (Bio-Rad). Fold changes in expression were calculated by the ΔΔ*C*_t_ method, using *ig7* as an endogenous control for mRNA expression. Fold changes are expressed normalized to vegetatively growing cells at 0 h cultivation.

### Proteomics

#### Sample preparation

Protein sample preparation was carried out using 10 × 10^6^ cells using an iST 8X kit (PreOmics), according to the manufacturer’s recommendation. All samples used for data-dependent acquisition (DDA) and data-independent acquisition (DIA) analyses were spiked with index retention time (iRT) kit peptides (Biognosys), according to the manufacturer’s instructions.

#### Construction of DIA spectral library

Spectral libraries were generated by Spectronaut version 10.0 using MaxQuant results as an input^55^. Fifteen shotgun (DDA) runs (using 2 or 3 biological replicates from each biological conditions) were acquired using a Q Exactive Plus instrument, and data were searched using MaxQuant (version 1.6.1.0). The spectral library was constructed using an FDR cut-off of 1% and a minimum and maximum of 3 and 6 fragment ions, respectively, and protein grouping was performed according to MaxQuant search results.

#### Mass spectrometric acquisition

The general nanoLC–MS setup was similar to that previously described^55^, with minor modifications. A Q Exactive Plus mass spectrometer (ThermoFisher) and an Easy nanoLC-1200 (ThermoFisher) were used for both DDA and DIA experiments. For the chromatographic separation of peptides, 4 μg peptide digest was analysed at 50 °C (controlled by Sonation column oven) on a 50-cm in-house packed fused-silica emitter microcolumn (75 μm inner diameter × 360 μm outer diameter SilicaTip PicoTip; New Objective) packed with 1.9-μm reverse-phase ReproSilPur C18-AQ beads (Dr. Maisch). Peptides were separated by a 4-h linear gradient of 5–80% (80% acetonitrile, 0.1% formic acid) at a constant flow rate of 300 nl min^−1^. For top 12 DDA acquisition, the ‘fast’ method from a previous publication^56^, was adopted, and DIA acquisition included a single MS1 survey scan at 35,000 resolution followed by 21 DIA windows^55^ (Supplementary Table 2).

#### Data analysis

DDA mass spectrometry raw files were analysed by MaxQuant software (version 1.6.1.0), and peak lists were searched against the *D. discoideum* UniProt FASTA database (version June 2018) concatenated with an in-house contaminant protein database by the Andromeda search engine embedded in MaxQuant^57,58^. The MS2-based label-free quantification was carried out by analysing DIA raw data using Biognosys Spectronaut (version 10.0) software using default parameters as previously described^55^, with minor modifications. In brief, the decoy method was set to ‘mutated’, data extraction and extraction window were set to ‘dynamic’ with correction factor 1, identification was set to ‘normal-distribution p-value estimator’ with *q*-value cut-off of 0.1, and the profiling strategy was set to ‘iRT profiling’ with *q*-value cut-off of 0.01. Ultimately, protein quantity was set to ‘Average precursor quantity’ and smallest quantitative unit was set to ‘Precursor ion’ (summed fragment ions). For statistical testing and identification of deregulated proteins in all approaches, a two-sample Student’s *t*-test was used to identify differentially expressed proteins filtered to 1% FDR.

### Assay for activity of mitochondrial aconitase

Twenty million cells per condition were plated in 10 ml HL5, development buffer, development buffer + cysteine or development buffer + GSH, in a 10-cm tissue-culture-treated dish. The supernatant was removed and cells were collected in ice-cold PBS, and homogenized in 150 μl assay buffer. Samples were centrifuged at 20,000*g* for 15 min at 4 °C, and the pellet was dissolved in 50 μl and sonicated for 20 s. The supernatant was collected, and mitochondrial aconitase activity was assayed using the BioVision aconitase activity colorimetric assay kit (BioVision), according to the manufacturer’s instructions.

### Protein synthesis assay

*Dictyostelium discoideum* were cultured in shaking culture in low fluorescence axenic LoFlo medium (ForMedium) overnight before the experiment. Two hundred thousand cells per condition were plated in 100 μl LoFlo medium in a 96-well tissue-culture-treated plate, and allowed to adhere for 1 h. The culture medium was then carefully removed and changed to the medium of interest (development buffer alone or supplemented with indicated nutrients or drugs), or replaced with fresh LoFlo medium, and cells were incubated for the indicated times. Protein synthesis was assayed using the Click-iT Plus OPP Alexa Fluor 647 protein synthesis assay kit (Molecular Probes), according to the manufacturer’s instructions. In brief, 30 min before the end of the treatment, cells were collected, disaggregated and cultured with 20 μM Click-iT OPP and Live/Dead Blue in PBS for 30 min. Cells were washed using 1× Perm/Wash Buffer (BD Biosciences) and fixed for 20 min at 4 °C in Fixation/Permeabilization solution (BD Biosciences). Cells washed using 1× Perm/Wash Buffer and incubated with 100 μl of the Click-iT Plus OPP reaction cocktail, prepared according to the manufacturer’s instructions, for 30 min. Cells were rinsed with Click-iT Reaction Rinse Buffer, and were collected using the Fortessa flow cytometer (BD Biosciences). Analysis was performed using FlowJo software (TreeStar).

### siRNA knockdown

In brief, 1 μl DharmaFect-1 (GE Healthcare) per well of a 24-well plate was mixed with 49 μl development buffer, and incubated for 5 min at room temperature. In a separate tube, 1 μl (100 nmol) *nfs1*-targeting or scrambled siRNA was made to a total volume of 50 μl with developing buffer and incubated for 5 min at room temperature. Tubes containing DharmaFect-1 and siRNA mixes were mixed and incubated for 20 min at room temperature. Then, 100 μl of this mixture was added to each well of a 24-well plate. One million vegetatively growing *D. discoideum* per well were resuspended in 400 μl of antibiotic-free HL5 medium and added to the 100 μl transfection mixture already in the well of the 24-well plate, for a total volume per well of 500 μl. Cells were incubated overnight before medium was changed to antibiotic-free HL5, development buffer or development buffer supplemented with cysteine, and further analysis was performed. For cells that were to be analysed by flow cytometry, LoFlo medium was used in place of HL5 at all steps.

### Mitochondrial isolation

Mitochondria were isolation according to a previously described protocol^59^. One hundred million cells per condition were plated in 50 ml of the appropriate medium in two 15-cm tissue-culture-treated plates (25 ml per plate). *Dictyostelium* were washed from the plate using HL5, development buffer or development buffer + cysteine, and centrifuged at 600*g* for 5 min at 4 °C. The supernatant was discarded, and cell pellets were resuspended in cold PBS before a further centrifugation at 600*g* for 5 min at 4 °C. The supernatant was discarded and the cell pellet was suspended in 2 ml ice-cold IB_cells_-1 (225 mM mannitol, 75 mM sucrose, 0.1 mM EGTA, 30 mM Tris-HCl pH 7.4, adjusted to pH 6.5). Cells were homogenized at 2,000 rpm using a Teflon pestle and pre-cooled glassware. One hundred strokes were sufficient to disrupt the majority of the cells. The homogenate was centrifuged at 600*g* for 5 min at 4 °C. The supernatant was centrifuged again at 600*g* for 5 min at 4 °C, and the pellet, containing unbroken cells and nuclei, was discarded. The supernatant was collected and centrifuged at 7,000*g* for 10 min at 4 °C. The supernatant from this step, containing lysosomes and microsomes, was discarded and the pellet was resuspended in 1 ml ice-cold IB_cells_-2 (225 mM mannitol, 75 mM sucrose, 30 mM Tris-HCl pH 7.4, adjusted to pH 6.5). This mitochondrial suspension was centrifuged at 7,000*g* for 10 min at 4 °C. The supernatant was discarded and the mitochondrial pellet was resuspended in 1 ml ice-cold IB_cells_-2 and centrifuged at 10,000*g* for 10 min at 4 °C. Mitochondrial protein concentration was determined by Qubit and mitochondria were resuspended in mitochondrial resuspension buffer (250 mM mannitol, 5 mM HEPES pH 7.4, 0.5 mM EGTA, adjusted to pH 6.5) at a concentration of 10 μg ml^−1^.

### Seahorse analysis of isolated mitochondria

Forty μg of mitochondria were loaded per well of a Seahorse XFp plate in 40 μl of mitochondrial assay buffer (MAS: 220 mM d-mannitol, 70 mM sucrose, 10 mM KH_2_PO_4_, 5 mM MgCl_2_, 2 mM HEPES, 1 mM EGTA, 0.2% w/v fatty acid-free BSA, pH 7.2)^60^ containing 10 mM malate, 10 mM glutamate, 4 mM ADP and 1,500 μM BHAM. The plate was centrifuged at 2,000*g* for 20 min at 4 °C. Then, 110 μl MAS containing malate, glutamate, ADP and BHAM was added to each well. Rotenone, succinate (10 mm), antimycin A or a combination of ascorbate (10 mM) and TMPD (100 μM) were injected as indicated.

### GSH and GSSG quantification

GSH and total glutathione were quantified using the GSH-Glo glutathione assay (Promega), according to the manufacturer’s instructions. Two hundred thousand cells per condition were plated in 100 μl HL5 medium in a 96-well tissue-culture-treated plate, and allowed to adhere for 1 h. The culture medium was then carefully removed and changed to the medium of interest (development buffer alone or supplemented with indicated nutrients or drugs), or replaced with fresh HL5 medium, and cells were incubated for the indicated times To detect GSH, the culture medium was removed, 50 μl of 1× GSH-Glo reagent was added to each well, and the plate was incubated at room temperature for 30 min. To detect total glutathione, the reducing agent TCEP was added to 1× GSH-Glo reagent at a concentration of 1 mM, to reduce GSSG to GSH. Fifty μl of reconstituted luciferin detection reagent was added to each well, and the plate was incubated in the dark for 15 min. Ninety μl of each sample was transferred to a white, opaque luminometer plate and luminescence was measured using a TriStar plate reader (Berthold Technologies). GSH and total glutathione concentrations were calculated from a GSH standard curve, and GSSG levels were calculated by subtracting GSH from total glutathione. The redox potential (*E*_h_) of the GSSG–GSH couple was calculated according to the Nernst equation.

### Statistical analysis

Statistical analysis was performed using Prism 7 software (GraphPad). Results are mean ± s.e.m. unless indicated otherwise; *n* represents independent biological replicates. Comparisons for two groups were calculated using unpaired two-tailed Student’s *t*-tests. MetaboAnalyst 4.0 was used for Small Molecule Pathway Database over-representation analysis of the incorporation of ^13^C^15^N-EEAs in starved *D. discoideum* (Fig. 3a). Statistical significance was calculated using a hypergeometric test, followed by the Holm–Bonferroni method to calculate adjusted *P* values. Exact *P* values are indicated in the figures. For proteomics pathway analysis, proteins that were altered with a *Q* value significance of <0.01 were subject to KEGG pathway analysis using STRING.

### Reporting summary

Further information on research design is available in the Nature Research Reporting Summary linked to this paper.

## Online content

Any methods, additional references, Nature Research reporting summaries, source data, extended data, supplementary information, acknowledgements, peer review information; details of author contributions and competing interests; and statements of data and code availability are available at 10.1038/s41586-021-03270-3.

## Supplementary information

Supplementary Information This file contains Supplementary Figs 1-2 and Supplementary Table 2.

Reporting Summary

Supplementary Table 1 Proteomics analysis of Starved Dictyostelium.

## Data Availability

All data that support the findings of this study are available within the Article and its Supplementary Information. Full scans of blots are provided in Supplementary Fig. 1. RNA-seq data have been deposited in the Gene Expression Omnibus (GEO) as the superseries GSE164011. This superseries contains RNA-seq datasets with accession number GSE164009, and a single-cell RNA-seq dataset with accession number GSE164010. The *Dictyostelium discoideum* genome assembly 2.7 (dicty_2.7, https://www.ncbi.nlm.nih.gov/assembly/GCF_000004695.1/) was used for RNA-seq analysis. The Human Metabolome Database (HMDB version 4.0, https://hmdb.ca/) was used for analysis of metabolite tracing data. The mass spectrometry proteomics data have been deposited to the ProteomeXchange Consortium via the PRIDE^61^ partner repository, with the dataset identifier PXD023404. Source data are provided with this paper.

## Source data

Source Data Fig. 1 Source Data Fig. 2 Source Data Fig. 3 Source Data Fig. 4 Source Data Extended Data Fig. 2 Source Data Extended Data Fig. 3 Source Data Extended Data Fig. 5 Source Data Extended Data Fig. 6 Source Data Extended Data Fig. 7 Source Data Extended Data Fig. 8 Source Data Extended Data Fig. 9 Source Data Extended Data Fig. 10

## Code availability

LC–MS and GC–MS metabolomics data were analysed using R code developed in-house, which is publicly available at https://gitlab.gwdg.de/joerg.buescher/metabolomics_scripts.
