# Supplementary material for: Sulfur sequestration promotes multicellularity during nutrient limitation
Source: Nature. 2021 Feb 24;591(7850):471–6. doi: 10.1038/s41586-021-03270-3 (PMC7969356; doi:10.1038/s41586-021-03270-3)
Supplement: Supplementary file 1 — This file contains Supplementary Figs 1-2 and Supplementary Table 2. [file 41586_2021_3270_MOESM1_ESM.pdf]

nature portfolio

https://doi.org/10.1038/s41586-021-03270-3

# Supplementary information

# Sulfur sequestration promotes multicellularity during nutrient limitation

In the format provided by the authors and unedited

**Sulfur sequestration promotes multicellularity during nutrient limitation**

Beth Kelly[1], Gustavo E. Carrizo[1], Joy Edwards-Hicks[1], David E. Sanin[1], Michal A. Stanczak[1], Chantal Priesnitz[2, 3], Lea J. Flachsmann[1], Jonathan D. Curtis[1], Gerhard Mittler[1], Yaarub Musa[1], Thomas Becker[4], Joerg M. Buescher[1], Erika L. Pearce[1*]

[1]Max Planck Institute for Immunobiology and Epigenetics, Freiburg 79108, Germany

[2]Institute of Biochemistry and Molecular Biology, ZMBZ, Faculty of Medicine, University of Freiburg

[3]Faculty of Biology, University of Freiburg

[4]Institute of Biochemistry and Molecular Biology, Faculty of Medicine, University of Bonn

*Correspondence to: pearce@ie-freiburg.mpg.de

## Table of Contents

**Supplementary Information**

**Supplementary Figure 1.**

Original source images for western blots 1

**Supplementary Figure 2**

Gating strategy to identify live, single *D. discoideum* cells 4

**Supplementary Table 2**

DIA windows scheduling used in this study 5

**Supplementary Table 1**

Proteomics analysis of Starved Dictyostelium
Provided as a separate Excel file

Fig. 1l

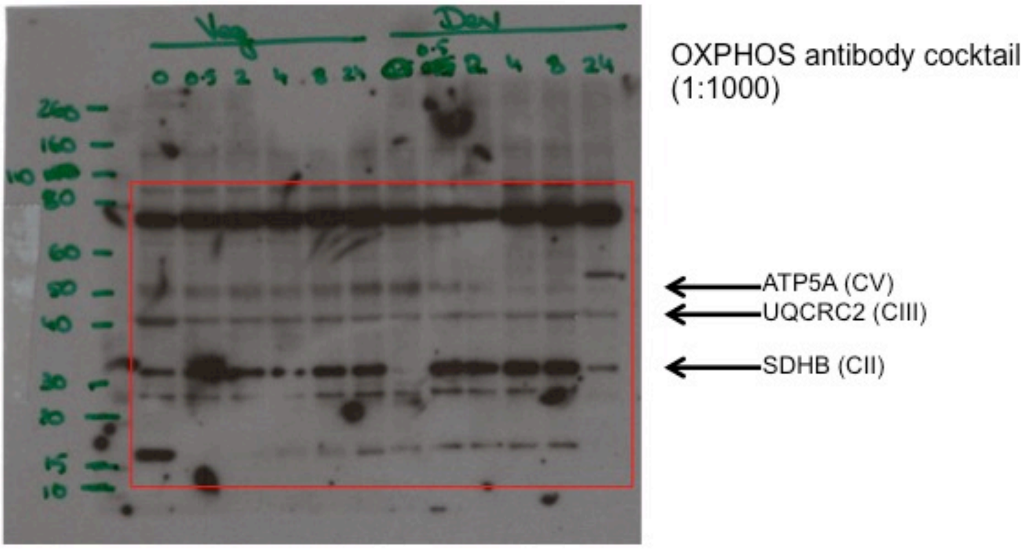


Fig. 2g

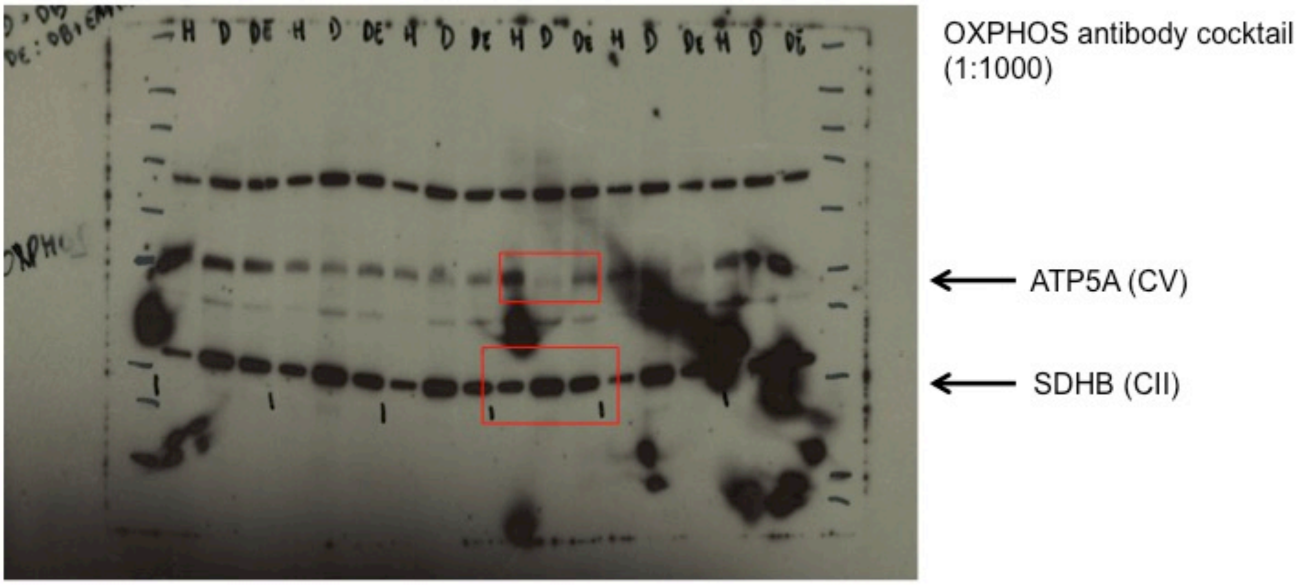


Fig. 3m

OXPHOS antibody cocktail (1:1000)

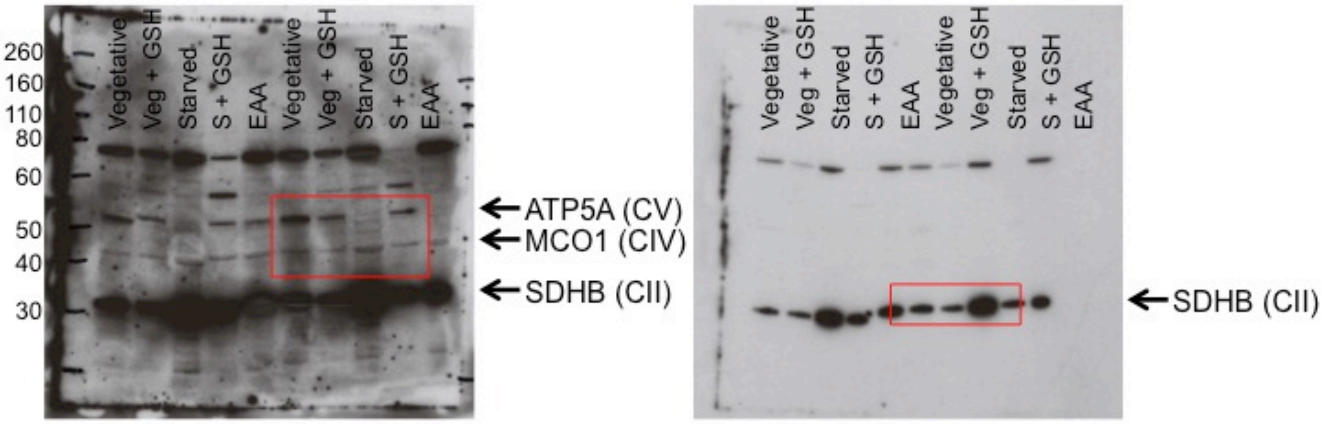


The above show two exposures of the same gel.

**Supplementary Figure 1. Original source images for western blots**

Extended Data Fig. 3e

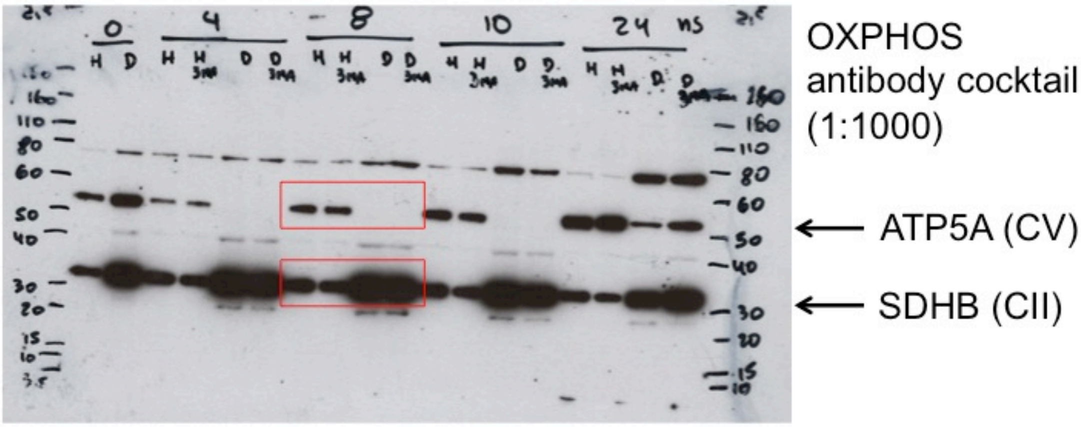


Extended Data Fig. 3g

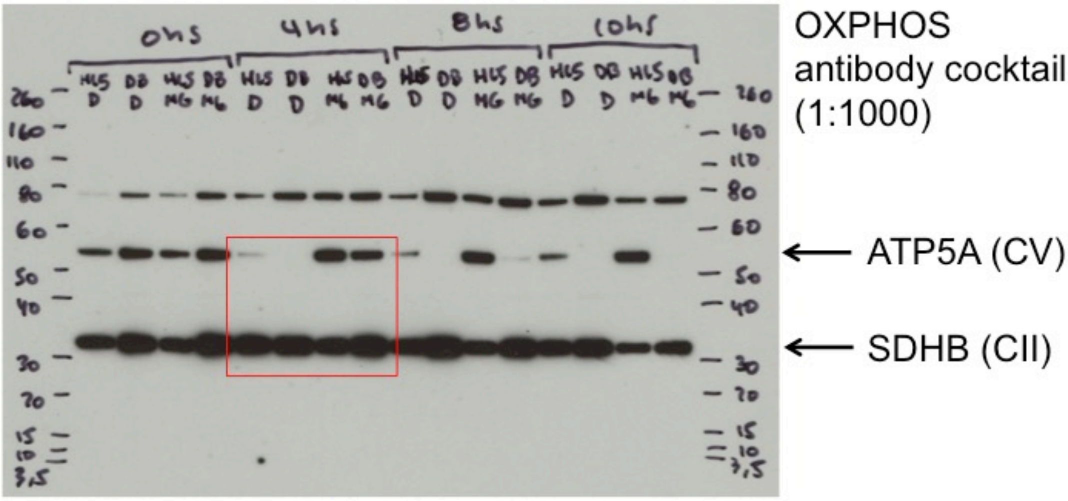


**Supplementary Figure 1. Original source images for western blots**

Extended Data Fig. 10e

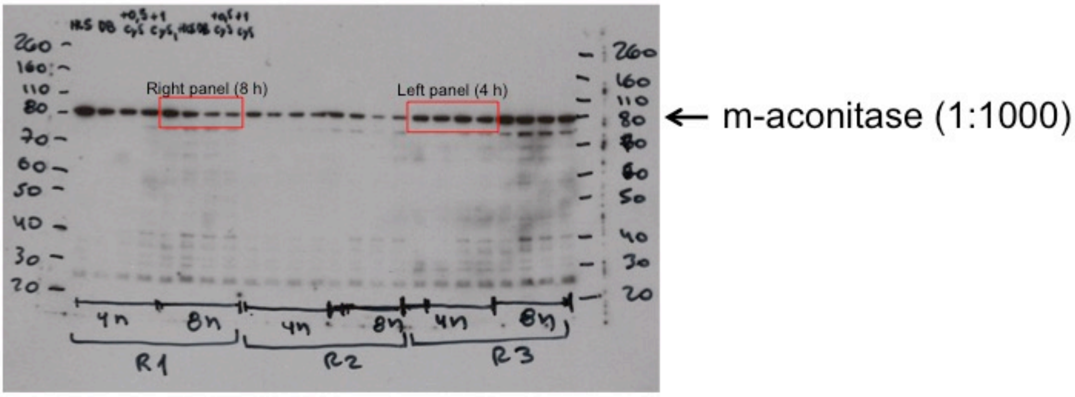


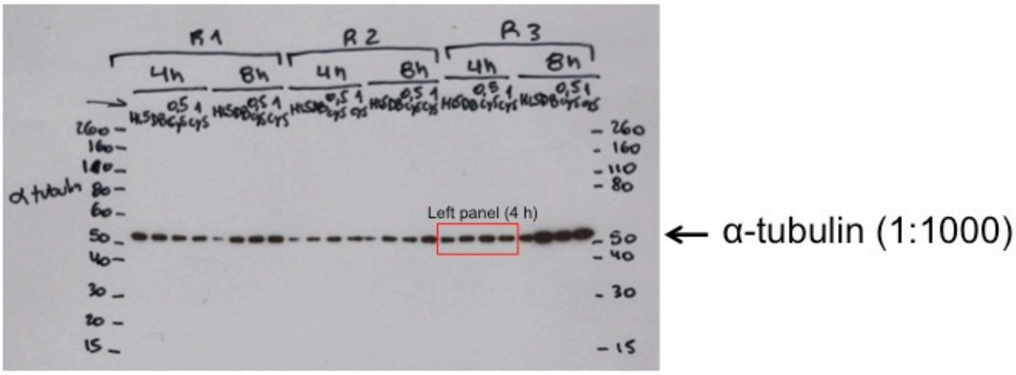


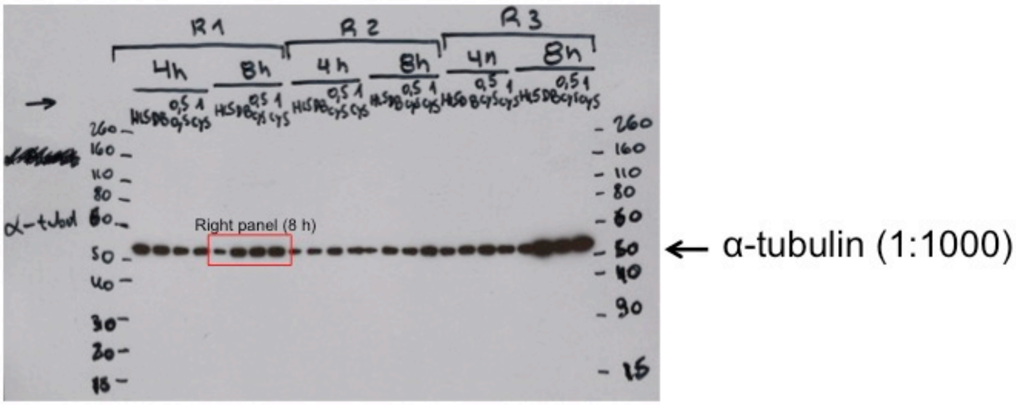


Samples and loading controls were run on the same gel.

**Supplementary Figure 1. Original source images for western blots**

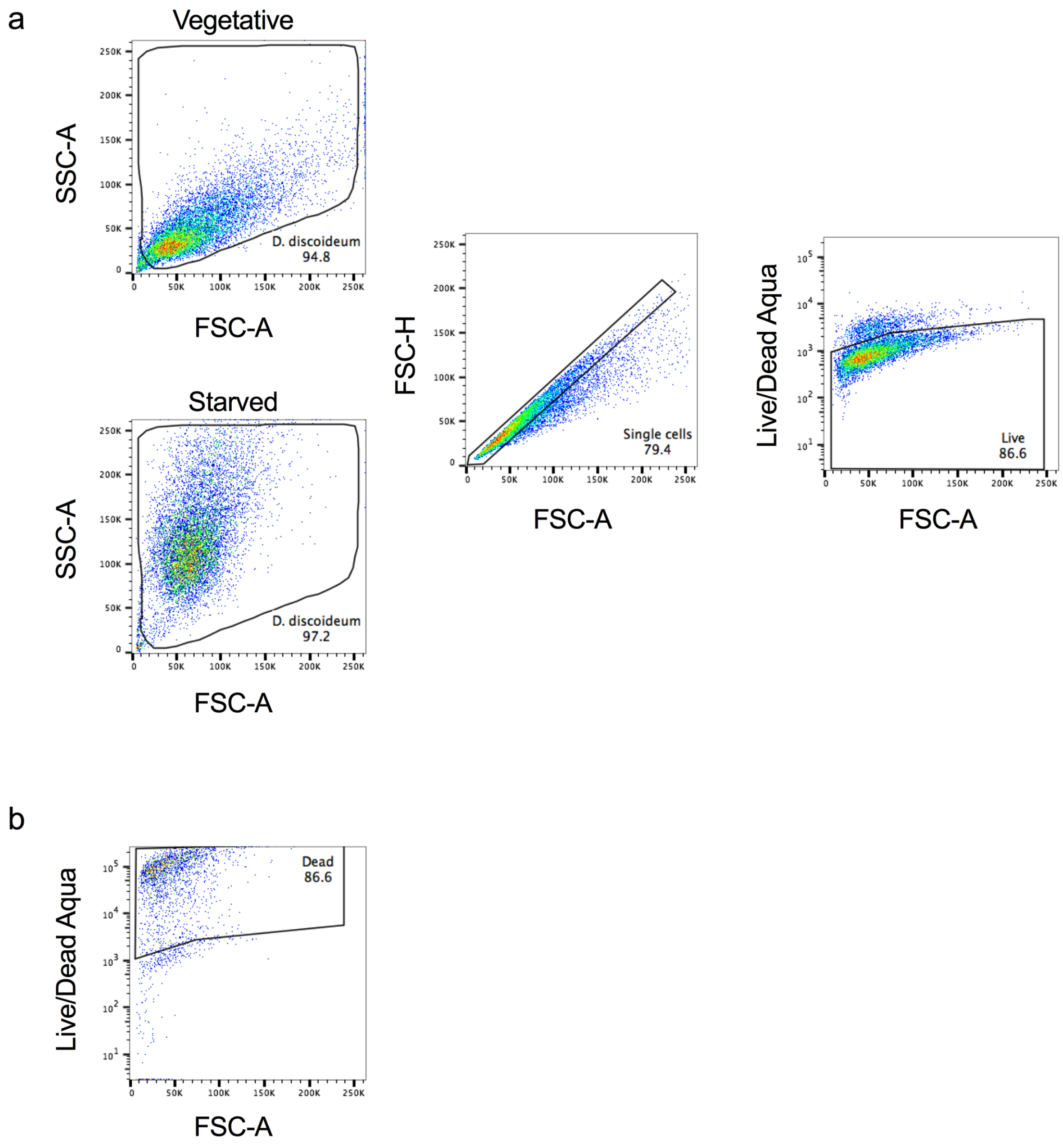


**Supplementary Figure 2. Gating strategy to identify live, single *D. discoideum* cells.** (a) Example flow cytometric gating strategy used to identify live, single, vegetative or starved *D. discoideum*. (b) Example of a positive control for dead cell staining.

Table S2

**DIA windows scheduling used in this study**

| | |
|---|---|
| Mass Range: | 400-1220 m/z |
| No. of DIA windows: | 21 |

| **Windows Scheduling** | | | | |
|---|---|---|---|---|
| **No.** | **Begin** | **End** | **Center** | **Window Width** |
| 1 | 400 | 426 | 413 | 26 |
| 2 | 425 | 451 | 438 | 26 |
| 3 | 450 | 476 | 463 | 26 |
| 4 | 475 | 501 | 488 | 26 |
| 5 | 500 | 526 | 513 | 26 |
| 6 | 525 | 551 | 538 | 26 |
| 7 | 550 | 576 | 563 | 26 |
| 8 | 576 | 601 | 588 | 26 |
| 9 | 600 | 631 | 615.5 | 31 |
| 10 | 630 | 661 | 645.5 | 31 |
| 11 | 660 | 691 | 675.5 | 31 |
| 12 | 690 | 721 | 705.5 | 31 |
| 13 | 720 | 771 | 745.5 | 51 |
| 14 | 770 | 821 | 795.5 | 51 |
| 15 | 820 | 871 | 845.5 | 51 |
| 16 | 870 | 921 | 895.5 | 51 |
| 17 | 920 | 971 | 945.5 | 51 |
| 18 | 970 | 1031 | 1000.5 | 61 |
| 19 | 1030 | 1091 | 1060.5 | 61 |
| 20 | 1090 | 1151 | 1120.5 | 61 |
| 21 | 1150 | 1220 | 1185 | 70 |

**Supplementary Table S2. DIA windows scheduling used in this study**
