# Supplementary material for: Sulfur sequestration promotes multicellularity during nutrient limitation
Source: Nature. 2021 Feb 24;591(7850):471–6. doi: 10.1038/s41586-021-03270-3 (PMC7969356; doi:10.1038/s41586-021-03270-3)
Supplement: Supplementary file 2 — Reporting Summary [file 41586_2021_3270_MOESM2_ESM.pdf]

nature research

Corresponding author(s): Erika Pearce

Last updated by author(s): Jan 5, 2021

Reporting Summary

Nature Research wishes to improve the reproducibility of the work that we publish. This form provides structure for consistency and transparency in reporting. For further information on Nature Research policies, see Authors & Referees and the Editorial Policy Checklist.

Statistics

For all statistical analyses, confirm that the following items are present in the figure legend, table legend, main text, or Methods section.

n/a	Confirmed	
☐	☒	The exact sample size (*n*) for each experimental group/condition, given as a discrete number and unit of measurement
☐	☒	A statement on whether measurements were taken from distinct samples or whether the same sample was measured repeatedly
☐	☒	The statistical test(s) used AND whether they are one- or two-sided<br>*Only common tests should be described solely by name; describe more complex techniques in the Methods section.*
☐	☒	A description of all covariates tested
☐	☒	A description of any assumptions or corrections, such as tests of normality and adjustment for multiple comparisons
☐	☒	A full description of the statistical parameters including central tendency (e.g. means) or other basic estimates (e.g. regression coefficient) AND variation (e.g. standard deviation) or associated estimates of uncertainty (e.g. confidence intervals)
☐	☒	For null hypothesis testing, the test statistic (e.g. *F*, *t*, *r*) with confidence intervals, effect sizes, degrees of freedom and *P* value noted<br>*Give P values as exact values whenever suitable.*
☒	☐	For Bayesian analysis, information on the choice of priors and Markov chain Monte Carlo settings
☒	☐	For hierarchical and complex designs, identification of the appropriate level for tests and full reporting of outcomes
☒	☐	Estimates of effect sizes (e.g. Cohen's *d*, Pearson's *r*), indicating how they were calculated

Our web collection on statistics for biologists contains articles on many of the points above.

Software and code

Policy information about availability of computer code

Data collection	Bulk RNA-sequencing data was collected using deepTools, STAR, featureCounts and the Dictyostelium discoideum genome assembly (2.7).<br>Single-cell RNA sequencing data was collected using Cell Ranger 2.2 (10X genomics) to genome build release 2-12.<br>Flow cytometric data was collected using FACSDiva (BD).<br>Discovery metabolomics (GC-MS) and metabolite quantification (LC-MS) data were collected using in-house R scripts that are publicly available (https://gitlab.gwdg.de/joerg.buescher/metabolomics_scripts).<br>Proteomics data was collected using Biognosys Spectronaut v 10.0 and MaxQuant v.1.6.1.0.
Data analysis	Bulk RNA-sequencing data was processed in R using DESeq2 and visualized using Morpheus (Broad Institute). RNA-seq pathway analysis was performed using String v11.0.<br>Single-cell RNA sequencing data was analyzed using Seurat v.3.<br>Flow cytometric data was analyzed using FlowJO V10 (TreeStar).<br>Metabolite tracing data was analyzed using AssayR and the Human Metabolomics Database (HMDB version 4.0). Metabolite pathway analysis was performed using the Kegg Mapper-Search Pathway tool and MetaboAnalyst 4.0. LC-MS or GC-MS metabolomics data was analysed using MassHunter B.07.01, or in-house R code that is publicly available (https://gitlab.gwdg.de/joerg.buescher/metabolomics_scripts).<br>Proteomics data was analyzed using Biognosys Spectronaut v10.0, MaxQuant software version 1.6.1.0 and the Dictyostelium discoideum UniProt FASTA database, version June 2018. Proteomics pathway analysis was performed using String v11.0.<br>Data analysis and statistical analysis was performed using Prism 7 software (GraphPad).

For manuscripts utilizing custom algorithms or software that are central to the research but not yet described in published literature, software must be made available to editors/reviewers. We strongly encourage code deposition in a community repository (e.g. GitHub). See the Nature Research guidelines for submitting code & software for further information.

Data

Policy information about availability of data

All manuscripts must include a data availability statement. This statement should provide the following information, where applicable:

- Accession codes, unique identifiers, or web links for publicly available datasets
- A list of figures that have associated raw data
- A description of any restrictions on data availability

RNA-seq data have been deposited in the Gene Expression Omnibus (GEO) as the superseries GSE164011. This superseries contains RNA-seq datasets with accession number GSE164009, and a scRNA-seq dataset with accession number GSE164010. The Dictyostelium discoideum genome assembly 2.7 (dicty_2.7, https://www.ncbi.nlm.nih.gov/assembly/GCF_000004695.1/) was used for RNA-seq analysis.
The mass spectrometry proteomics data have been deposited to the ProteomeXchange Consortium via the PRIDE partner repository with the dataset identifier PXD023404The Human Metabolome Database (HMDB version 4.0, https://hmdb.ca/) was used for analysis of metabolite tracing data.
Source data for Figures 1 - 4 and Extended Data Figures 2, 3, and 5 - 10, as well as full scans for all western blots, have been provided provided with the paper.

Field-specific reporting

Please select the one below that is the best fit for your research. If you are not sure, read the appropriate sections before making your selection.

☒ Life sciences ☐ Behavioural & social sciences ☐ Ecological, evolutionary & environmental sciences

For a reference copy of the document with all sections, see nature.com/documents/nr-reporting-summary-flat.pdf

Life sciences study design

All studies must disclose on these points even when the disclosure is negative.

Sample size	No statistical tests were used to determine sample size. Sample sizes were determined based on the standard in the field, and on the numbers required to achieve statistical significance using indicated statistics. Replication between biological samples was robust, so at least 3 biological replicates were used for each experiment.
Data exclusions	In very rare cases, a single biological replicate was excluded due to experimental error. All results are still drawn from a minimum of 3 independent biological replicates.
Replication	All experiments used at least 3 independent biological replicates. Biological replicates were taken from independently growing Dictyostelium discoideum cultures. Reported results were consistently replicated across multiple independent experiments and all replicates generated similar results.
Randomization	No randomization was performed as when comparing treated versus control conditions, both the control and the treated sample came from the same D. discoideum culture. This was then repeated using a minimum of two further independently growing D. discoideum cultures, so that each experiment involving multiple conditions or treatments was repeated in at least three independent biological replicates, originating from independently growing D. discoideum cultures. No in vivo studies were performed.
Blinding	Blinding was not required for this work. Samples were standardized and were treated in exactly the same way prior to treatment, conditions were well-controlled, and the results are quantitative, objective measures, not subject to bias or objective judgment. Thus, the integrity of the results is not impacted when running the study unblinded. All assessments were carried out using multiple independent replicates.

Reporting for specific materials, systems and methods

We require information from authors about some types of materials, experimental systems and methods used in many studies. Here, indicate whether each material, system or method listed is relevant to your study. If you are not sure if a list item applies to your research, read the appropriate section before selecting a response.

Materials & experimental systems

n/a	Involved in the study
☐	☒ Antibodies
☒	☐ Eukaryotic cell lines
☒	☐ Palaeontology
☒	☐ Animals and other organisms
☒	☐ Human research participants
☒	☐ Clinical data

Methods

n/a	Involved in the study
☒	☐ ChIP-seq
☐	☒ Flow cytometry
☒	☐ MRI-based neuroimaging

Antibodies

Antibodies used	1. Total OXPHOS Rodent WB Antibody Cocktail, Abcam, Cat. No. ab110413, Lot No. N3113<br>2. 12G10 anti-alpha tubulin-s, Supplied by Developmental Studies Hybridoma Bank (DSHB) at the University of Iowa, Cat. No. 12G10<br>3. anti-ATP5A [15H4C4] - mitochondrial marker (FITC), Abcam, Cat. No. ab119688, Lot No. GR3238328-13. anti-ATP5A [15H4C4] - mitochondrial marker (FITC), Abcam, Cat. No. ab119688, Lot No. GR3238328-1<br>4. anti-aconitase 2, Abcam, Cat. No. ab83528, Lot No. GR3289983-4<br>5. rabbit anti-mouse IgG (H+L) secondary antibody, HRP, ThermoFisher Scientific, Cat. No. 31450<br>6. goat rabbit IgG (H+L) secondary antibody, HRP, ThermoFisher Scientific, Cat. No. 31460
Validation	The Total OXPHOS Rodent WB Antibody Cocktail is guaranteed by the manufacturer (Abcam) for Western Blot detection of OXPHOS protein complexes in mouse, rat, cow, human and monkey. The detected complexes are well-conserved between Dictyostelium discoideum and these species, and the bands for these complexes appear at similar sizes in Dictyostelium.<br>The anti-ATP5A [15H4C4] - mitochondrial marker (FITC) is validated by Abcam for flow cytometry in multiple species.<br>The 12G10 anti-alpha tubulin-s is validated for Western blot in Dictyostelium by the Developmental Studies Hybridoma Bank (DSHB) at the University of Iowa. The anti-aconitase 2 antibody is guaranteed by the manufacturer (Abcam) for Western Blot in multiple species, including human, rat, cow, pig and S. cerevisiae.

Flow Cytometry

Plots

Confirm that:

☒ The axis labels state the marker and fluorochrome used (e.g. CD4-FITC).

☒ The axis scales are clearly visible. Include numbers along axes only for bottom left plot of group (a 'group' is an analysis of identical markers).

☒ All plots are contour plots with outliers or pseudocolor plots.

☒ A numerical value for number of cells or percentage (with statistics) is provided.

Methodology

Sample preparation	Suspensions of Dictyostelium discoideum were generated by gentle mechanical disruption. Samples were stained for 30 minutes in PBS and were protected from light throughout.
Instrument	Data were collected on LSR II or LSRFortessa (both BD).
Software	Data were collected using FACSDiva Software (BD).<br>Data were analyzed using FlowJo V10 Software.
Cell population abundance	No cell sorting was performed.
Gating strategy	Dictyostelium discoideum were identified and debris was excluded using FSC/SSC. Doublets were excluded using FSC-H/FSC-W. Live cells were identified using Live/Dead Blue, Live/Dead Aqua, or Live/Dead Near-IR (all ThermoFisher Scientific). Cellular and mitochondrial ROS production were identified using CellROX Deep Red and MitoSOX Red, respectively (both ThermoFisher Scientific). Protein synthesis was quantified by OPP-AF647 fluorescence. Cystine uptake was measured using BioTracker cystine-FITC live cell dye (Merck).

☒ Tick this box to confirm that a figure exemplifying the gating strategy is provided in the Supplementary Information.
